# Differences in the Epigenetic Regulation of Cytochrome P450 Genes between Human Embryonic Stem Cell-Derived Hepatocytes and Primary Hepatocytes

**DOI:** 10.1371/journal.pone.0132992

**Published:** 2015-07-15

**Authors:** Han-Jin Park, Young-Jun Choi, Ji Woo Kim, Hang-Suk Chun, Ilkyun Im, Seokjoo Yoon, Yong-Mahn Han, Chang-Woo Song, Hyemin Kim

**Affiliations:** 1 Department of Biological Sciences and Center for Stem Cell Differentiation, Korea Advanced Institute of Science and Technology, Daejeon, 305–701, Republic of Korea; 2 Department of Predictive Toxicology, Korea Institute of Toxicology, Daejeon, 305–343, Republic of Korea; 3 Department of Inhalation Research, Korea Institute of Toxicology, Jeollabuk-do, 580–185, Republic of Korea; 4 Human and Environmental Toxicology, School of Engineering, University of Science and Technology, Daejeon, 303–333, Republic of Korea; University of Tampere, FINLAND

## Abstract

Human pluripotent stem cell-derived hepatocytes have the potential to provide *in vitro* model systems for drug discovery and hepatotoxicity testing. However, these cells are currently unsuitable for drug toxicity and efficacy testing because of their limited expression of genes encoding drug-metabolizing enzymes, especially cytochrome P450 (CYP) enzymes. Transcript levels of major *CYP* genes were much lower in human embryonic stem cell-derived hepatocytes (hESC-Hep) than in human primary hepatocytes (hPH). To verify the mechanism underlying this reduced expression of *CYP* genes, including *CYP1A1*, *CYP1A2*, *CYP1B1*, *CYP2D6*, and *CYP2E1*, we investigated their epigenetic regulation in terms of DNA methylation and histone modifications in hESC-Hep and hPH. CpG islands of *CYP* genes were hypermethylated in hESC-Hep, whereas they had an open chromatin structure, as represented by hypomethylation of CpG sites and permissive histone modifications, in hPH. Inhibition of DNA methyltransferases (DNMTs) during hepatic maturation induced demethylation of the CpG sites of *CYP1A1* and *CYP1A2*, leading to the up-regulation of their transcription. Combinatorial inhibition of DNMTs and histone deacetylases (HDACs) increased the transcript levels of *CYP1A1*, *CYP1A2*, *CYP1B1*, and *CYP2D6*. Our findings suggest that limited expression of *CYP* genes in hESC-Hep is modulated by epigenetic regulatory factors such as DNMTs and HDACs.

## Introduction

Pluripotent stem cells (PSCs) and their derivatives will be valuable in regenerative medicine and for the development and discovery of new drugs. In particular, PSC-derived hepatocytes have many advantages over primary hepatocytes and hepatocellular carcinoma cell lines, such as their unlimited supply and better functionality, for the *in vitro* assessment of drug-induced hepatotoxicity[1–7]. Human PSCs can differentiate into hepatocytes that exhibit several liver-specific characteristics, such as the expression of hepatocyte marker genes, albumin (ALB) secretion, glycogen storage, and active cytochrome P450 (CYP) enzymes, which are representative of phase I enzymes in drug metabolism [2,8–12]. Although the homogeneity and functional properties of PSC-derived hepatocytes are continually improving, they cannot fully replicate drug metabolism in the liver at present [13]. Most studies have found that low mRNA levels and activities of CYP enzymes were detected in PSC-derived hepatocytes than in adult hepatocytes [13–15].

The developmental stage of the liver is closely correlated with the expression and activities of *CYP* genes [16–18]. One way in which the expression of *CYP* genes is controlled during development is epigenetic regulation [19], which refers to genomic modifications that can influence gene expression and cellular phenotypes but do not change the DNA sequence [20]. DNA methylation and histone modifications participate in the regulation of human *CYP* genes and this has mainly been reported in cancer [19,21,22]. Recent study proves that DNA methylation is associated with variations in hepatic gene expression between fetal and adult human liver [23]. Also, the difference in expression of epigenetic modifier genes, which are responsible for regulating histone and DNA modifications, represents between human embryonic stem cell (hESC)-derived hepatocytes (hESC-Hep) and primary hepatocytes [24]. However, epigenetic regulation of *CYP* genes during liver development is poorly understood. In this study, we investigated the reduced expression of *CYP* genes in hESC-Hep and epigenetic differences in regulatory regions around the transcription start sites (TSS) of *CYP* genes between hESC-Hep and human primary hepatocytes (hPH). Some *CYP* genes were regulated by inhibition of DNA methyltransferases (DNMTs) and histone deacetylases (HDACs) during the differentiation of hESCs into hepatocytes.

## Results

### Reduced expression of drug-metabolizing enzyme (DME) genes in hESC-Hep

**Fig 1:**
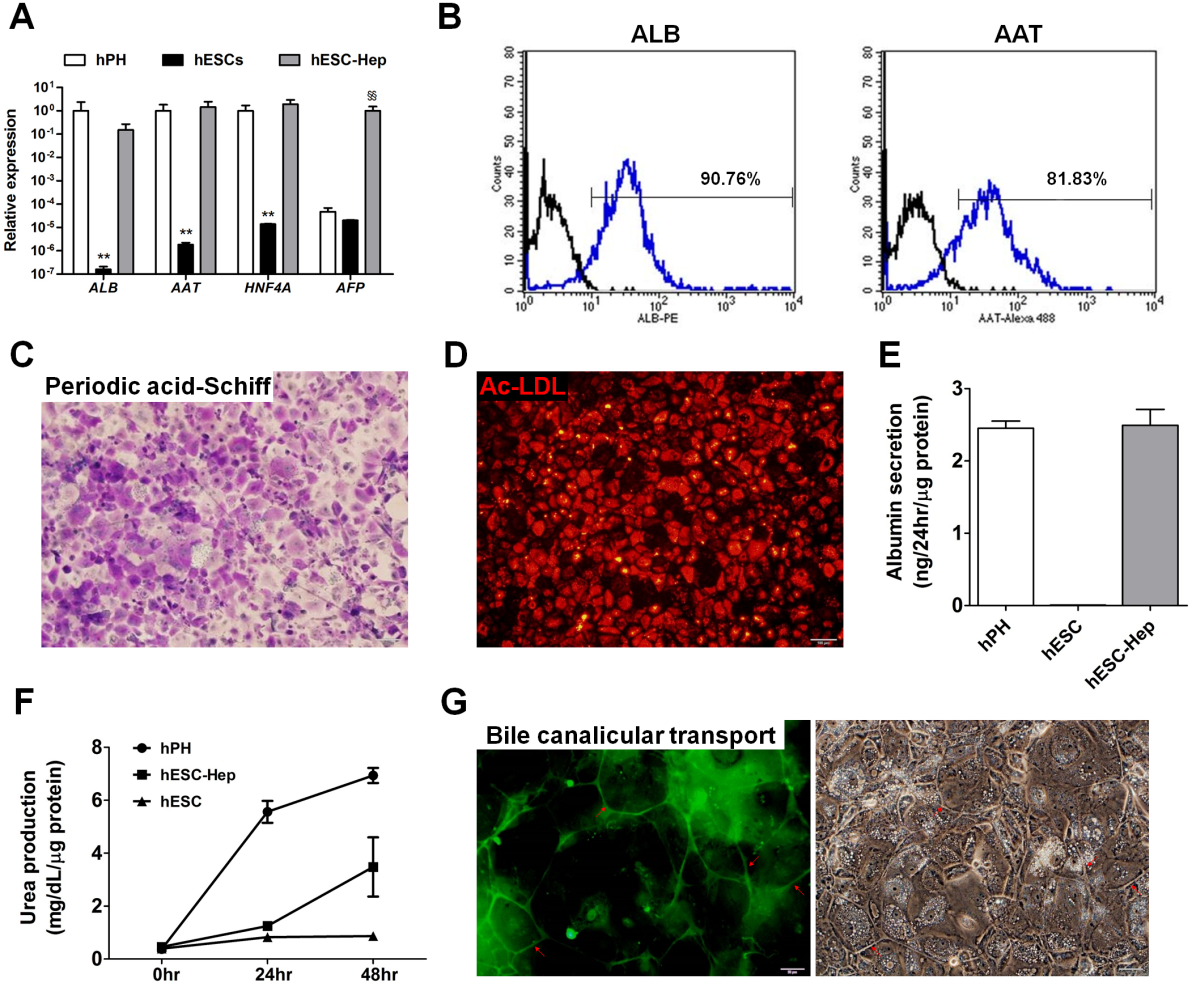
Differentiation of human embryonic stem cells (hESCs) into hepatocytes *in vitro*. (A) Expression of hepatocyte marker genes. Genes were examined by real-time RT-PCR in hPH, hESCs, and hESC-Hep (day 20 of differentiation). Data represent mean ± SD from three independent experiments. ** p<0.01, significant values in comparison with hPH; ^§§^ p<0.01, significant values in comparison with hESC-Hep (ANOVA followed by Dunn’s multiple comparison test). (B) Percentages of albumin (ALB)-positive and α-1-antitrypsin (AAT)-positive cells among hESC-Hep. Fluorescence-activated cell sorting analysis was performed 20 days after the onset of differentiation. Black line: isotype control, blue line: primary antibody. (C) Glycogen storage in hESC-Hep. Periodic acid-Schiff staining of glycogen was performed at day 20 of differentiation. Stored glycogen (purple) was observed in the cytoplasm. Nuclei (light blue) were counterstained with hematoxylin. The scale bar represents 100 μm. (D) Acetylated-low-density lipoprotein (Ac-LDL) uptake by hESC-Hep. The ability of cells to take up Ac-LDL was examined at day 20 of differentiation. The scale bar represents 100 μm. (E) ALB secretion from hESC-Hep. The ALB concentration was measured in the conditioned media of hESCs (day 0), hESC-Hep (day 20), and hPH by an enzyme-linked immunosorbent assay using an anti-human ALB antibody. (F) Urea production by hESC-Hep. The amount of urea secreted by hESCs (day 0), hESC-Hep (day 20), and hPH was examined at 0, 24, and 48 hours. (G) Activity of bile canalicular transporter in hESC-Hep. Red arrows indicate cleaved 5-(and-6)-carboxy-2’,7’-dichlorofluorescein diacetate, which was excreted into the bile canalicular spaces of cells. The scale bars represent 50 μm.

hESCs were differentiated into hepatocytes via definitive endoderm (DE) as detailed in S1 Fig. Approximately 99% of cells differentiated into CXCR4-positive DE cells on day 5 (D5, S2A Fig). At the end of differentiation, Expression of the hepatocyte markers ALB, α-1-antitrypsin (AAT), α-fetoprotein (AFP), and hepatocyte nuclear factor 4 α (HNF4A) was detected in hESC-Hep at the transcript and protein levels (Fig 1A and S2B Fig). Approximately 90% and 81% of hESC-Hep expressed ALB and AAT, respectively, according to fluorescence-activated cell sorting (FACS) (Fig 1B). hESC-Hep could store glycogen in the cytoplasm and take up acetylated-low-density lipoprotein (Fig 1C and 1D). hESC-Hep could also secrete ALB into the culture media and synthesize urea like hPH (Fig 1E and 1F). Moreover, bile canaliculi formation and function in hESC-Hep were verified using 5-(and-6)-carboxy-2’,7’-dichlorofluorescein diacetate (carboxy-DCFDA). Cells were incubated with carboxy-DCFDA, which was internalized by hepatocytes, cleaved by intracellular esterases, and excreted from bile canaliculi. 5-(and-6)-Carboxy-2’,7’-dichlorofluorescein accumulated in bile canaliculi between adjacent cells (Fig 1G). These results demonstrate that hESC-Hep have cellular and molecular characteristics of hepatocytes.

**Fig 2:**
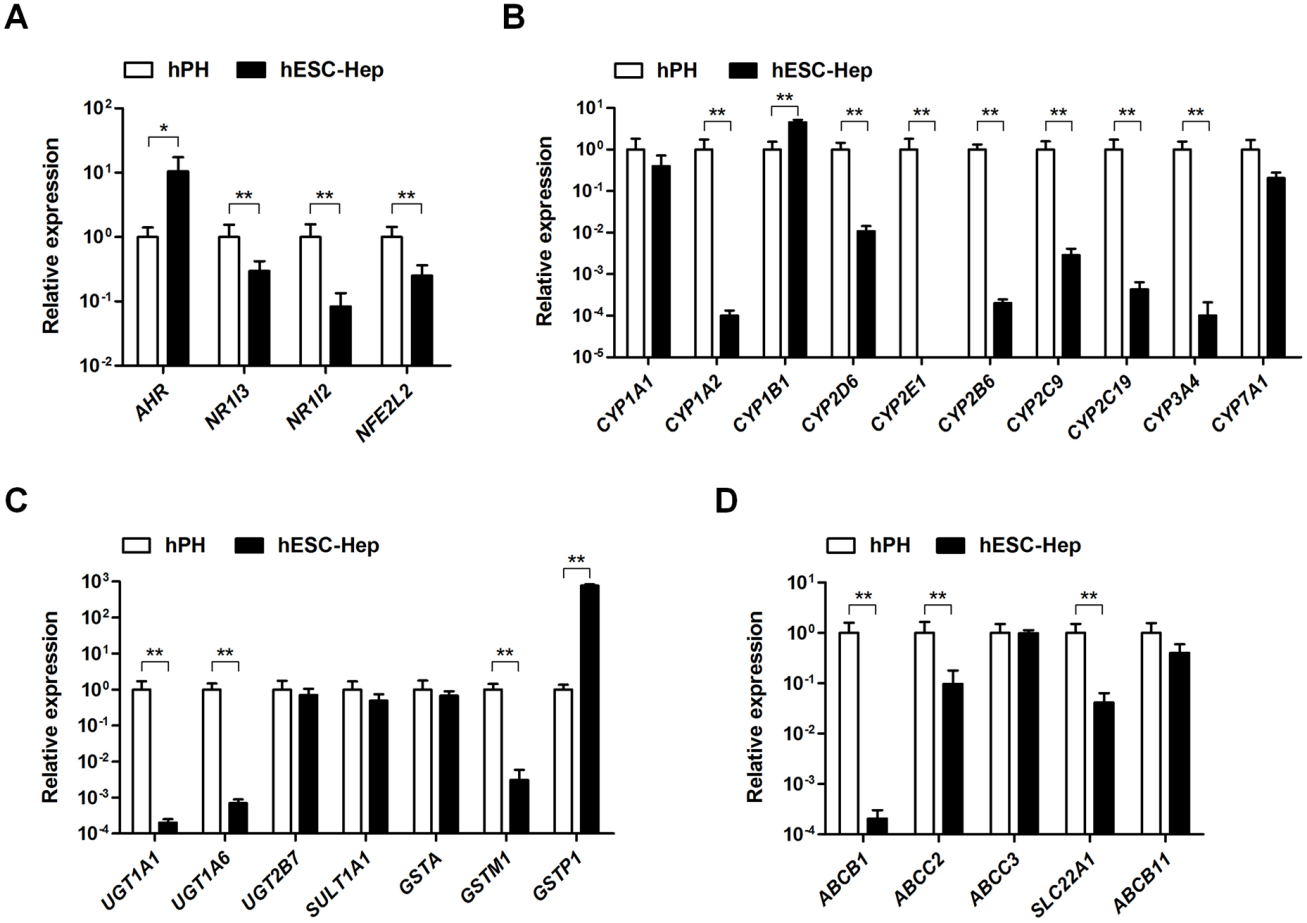
Gene expression levels of drug-metabolizing enzymes. Expression of genes encoding nuclear receptors (A), phase I enzymes (B), phase II enzymes (C), and phase III transporters (D) was examined by real-time RT-PCR in human primary hepatocytes (hPH) and human embryonic stem cell-derived hepatocytes (hESC-Hep, day 20). Data represent mean ± SD from three independent experiments. * p<0.05, ** p<0.01, significant values in comparison with hPH (t-test followed by Wilcoxon matched pairs test).

Next, we examined the expression of DME genes in hESC-Hep. The nuclear receptor *NR1I3* (CAR), *NR1I2* (PXR), and *NFE2L2* (NRF2) was significantly expressed in hESC-Hep than in hPH, whereas *AHR* was highly expressed compared to hPH (Fig 2A). Expression of most DME genes, including those encoding phase I enzymes, phase II enzymes, and phase III transporters, was lower in hESC-Hep than in hPH (Fig 2B–2D). In terms of phase I enzymes, expression of *CYP1A1*, *CYP1B1*, and *CYP7A1* in hESC-Hep was similar to or higher than that in hPH (Fig 2B). However, *CYP1A2*, *CYP2B6*, *CYP2C9*, *CYP2C19*, *CYP2D6*, *CYP2E1*, and *CYP3A4* were weakly expressed in hESC-Hep compared to hPH (Fig 2B). Transcript levels of phase II enzymes, including *UGT2B7*, *SULT1A1*, *GSTA*, and *GSTP1*, in hESC-Hep were similar to or higher than the levels in hPH (Fig 2C). hESC-Hep expressed phase III transporters, such as those encoded by *ABCC3* and *ABCB11* (Fig 2D). These results demonstrate that hESC-Hep have a limited ability to metabolize drugs because most DME genes, especially those encoding major CYP enzymes such as CYP1A2, CYP2D6, and CYP3A4, were not expressed during the differentiation of hESCs into hepatocytes *in vitro*.

### Differences in the epigenetic modifications of *CYP* genes between hESC-Hep and hPH

In this study, we showed that expression of major CYP genes such as *CYP1A2*, *CYP2C9*, *CYP2C19*, *CYP2D6*, *CYP3A4*, and *CYP2E1* was extremely low, whereas *CYP1A1* and *1B1* genes were similar and highly expressed compared to hPH, respectively (Fig 2B). To investigate whether epigenetic modifications modulate *CYP* gene expression during the differentiation of hESCs into hepatocytes *in vitro*, we studied combinatorial roles of DNA methylation and histone modifications at regulatory regions around the TSS of CYP enzymes in hESC-Hep and hPH. We selected five CYP genes including *CYP1A1*, *CYP1A2*, *CYP1B1*, *CYP2D6*, and *CYP2E1*, which have CpG islands in regulatory regions. However, *CYP2C9*, *CYP2C19*, and *CYP3A4* do not contain CpG islands.

**Fig 3:**
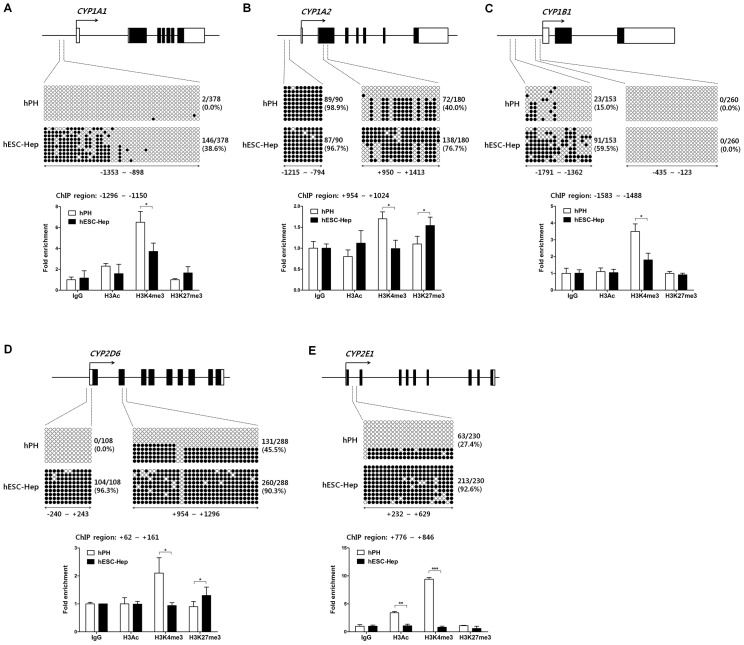
DNA methylation and histone modifications in regulatory regions of *CYP* genes. Each diagram shows the locations of the sites of *CYP1A1* (A), *CYP1A2* (B), *CYP1B1* (C), *CYP2D6* (D), and *CYP2E1* (E) within gene promoters, which were examined by bisulfite sequencing and chromatin immunoprecipitation (ChIP). The methylation status of CpG dinucleotides in target regions was examined in hPH and hESC-Hep (day 20) by bisulfite sequencing (upper panel). Each row represents the methylation status of each CpG in one bacterial clone. A series of 9–10 clones is shown. Black circles represent metyhylated CpG sites while white circles represent unmethylated CpG sites. Numbers indicate nucleotide positions in relation to the transcription start site (TSS, +1). ChIP analysis of histone modifications including two active histone marks H3Ac and H3K4me3 and one repressive histone mark H3K27me3 in hPH and hESC-Hep is shown (lower graph). Data validated by real-time PCR are presented as fold enrichment of precipitated DNA associated with a given histone modification relative to a 100-fold dilution of input chromatin. Data represent mean ± SD from two independent experiments. * p<0.05, ** p<0.01, *** p<0.001, significant values in comparison with hPH (t-test followed by Wilcoxon matched pairs test).

Expression of *CYP1A1* and *CYP1B1* in hESC-Hep was similar to or higher than that in hPH, whereas *CYP1A2* was slightly expressed in hESC-Hep (Fig 2B). Bisulfite sequencing of the *CYP1A1* promoter region, which contains 42 CpG dinucleotides, revealed the methylation frequency was 0.0% and 38.6% in hPH and hESC-Hep, respectively (Fig 3A, upper panel). In hESC-Hep, distribution of methylated CpG sites in *CYP1A1* was located at position -1353 to -1100 relative to TSS, which includes 18 CpG dinucleotides (Fig 3A, upper panel). In this region, enrichment of active histone mark histone H3 trimethylated at lysine 4 (H3K4me3) in hPH was higher than that in hESC-Hep (Fig 3A, lower panel). By contrast, unmethylated CpG sites (-1099 to -898 relative to TSS) represented similar enrichment patterns of histone modifications between hPH and hESC-Hep (S3A Fig). The promoter region of the *CYP1A2* gene, which contains 10 CpG dinucleotides, was completely methylated in hPH and hESC-Hep (Fig 3B, upper left panel). However, the CpG island in the gene body region, which contains 20 CpG dinucleotides, had a methylation frequency of 40.0% and 76.7% in hPH and hESC-Hep, respectively (Fig 3B, upper right panel). H3K4me3 in the gene body region of *CYP1A2* likely modulated expression of this gene in hPH (Fig 3B, lower graph). The presence of repressive histone mark histone H3 trimethylated at lysine 27 (H3K27me3) in hESC-Hep was associated with down-regulation of *CYP1A2* transcription (Fig 3B, lower graph). The methylation frequency at -1791 to -1362 relative to TSS of *CYP1B1*, which includes 17 CpG dinucleotides, was 15.0% and 59.5% in hPH and hESC-Hep, respectively (Fig 3C, upper left panel). This region was mainly occupied by H3K4me3 in hPH, while levels of H3Ac and H3K27me3 were similar to those of the IgG controls in hPH and hESC-Hep (Fig 3C, lower graph). However, the CpG island in close to TSS (-435 to -123), which contains 26 CpG dinucleotides, was completely demethylated in hPH and hESC-Hep (Fig 3C, upper right panel). In this region, H3K4me3 was enriched in hPH and hESC-Hep, although H3Ac was only found in hPH (S3B Fig). Therefore, these results indicate that expression levels of *CYP1A1*, *CYP1A2*, and *CYP1B1* are regulated by DNA methylation and histone modifications in the regulatory region.

The transcript levels of *CYP2D6* and *CYP2E1* were low in hESC-Hep (Fig 2B). The methylation frequency at the *CYP2D6* promoter region, which contains 12 CpG dinucleotides, was 0.0% and 96.3% in hPH and hESC-Hep, respectively (Fig 3D, upper left panel). In the gene body region of *CYP2D6*, which contains 32 CpG dinucleotides, the methylation frequency was 45.5% and 90.3% in hPH and hESC-Hep, respectively (Fig 3D, upper right panel). The promoter region of *CYP2D6* was enriched in H3K4me3 in hPH, and silencing of this gene in hESC-Hep was likely governed by H3K27me3 (Fig 3D, lower graph). Bisulfite sequencing of the *CYP2E1* gene body region, which contains 23 CpG dinucleotides, revealed a methylation frequency of 27.4% and 92.6% in hPH and hESC-Hep, respectively (Fig 3E, upper panel). H3Ac and H3K4me3 were enriched in the gene body region of *CYP2E1* in hPH, while the levels of H3K27me3 were similar to those of IgG controls in hPH and hESC-Hep (Fig 3E, lower graph). These results show that inhibitory epigenetic regulation of *CYP2D6* and *CYP2E1* in hESC-Hep is associated with the transcriptional inactivation of these genes. Therefore, our data represents the epigenetic differences in the regulatory regions of five *CYP* genes between hESC-Hep and hPH. Reduced transcription of *CYP1A2*, *CYP2D6*, and *CYP2E1* in hESC-Hep may be influenced by DNA methylation and histone modifications.

### Transcriptional regulation of *CYP* genes by inhibition of DNMTs and HDACs

**Fig 4:**
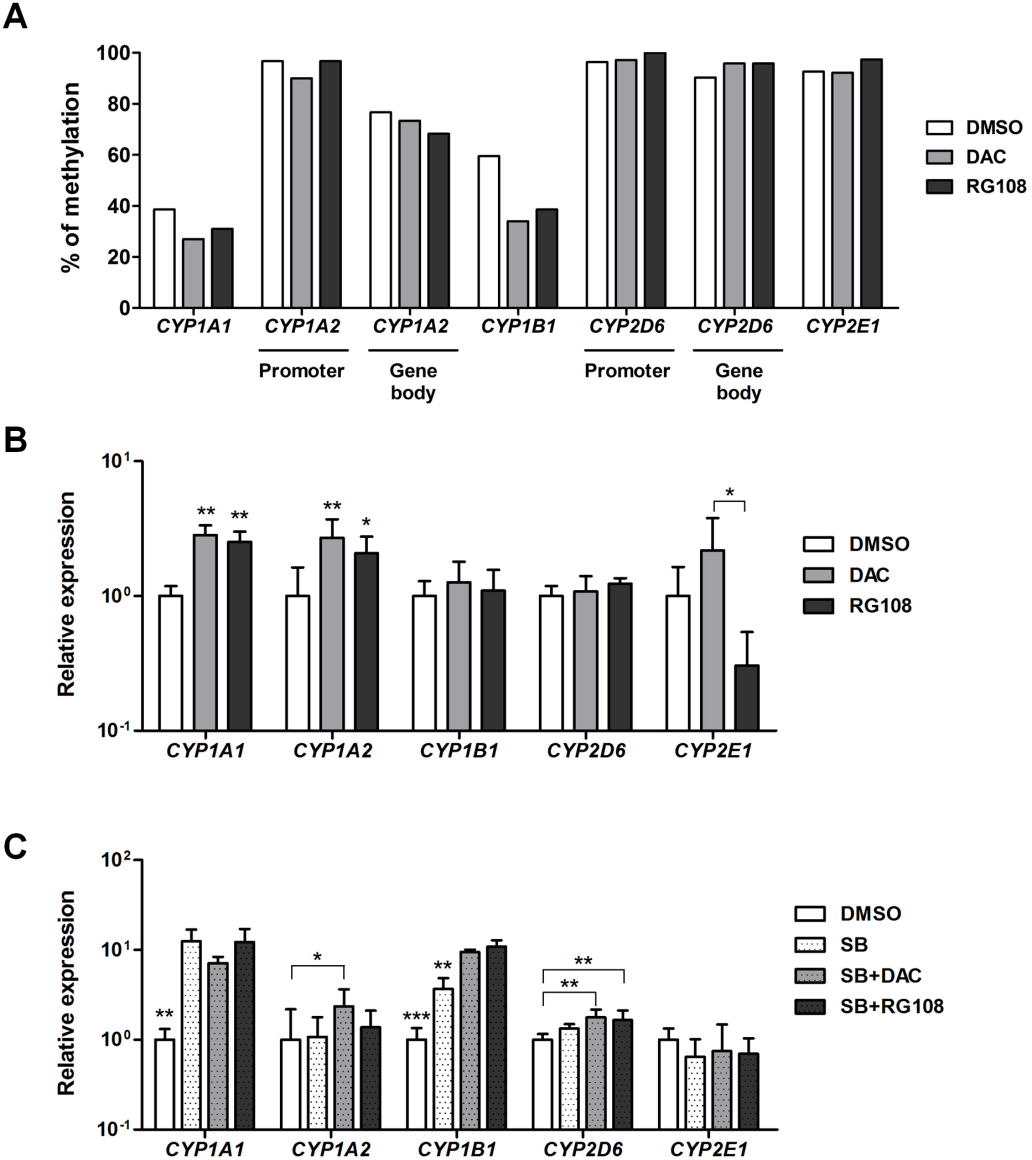
Transcriptional regulation of *CYP* genes by inhibition of DNA methyltransferases (DNMTs) and histone deacetylases (HDACs) during hepatic differentiation. (A) Methylation frequencies in the promoter and gene body regions of *CYP* genes were examined by bisulfite sequencing in hESC-Hep treated with DMSO or a DNMT inhibitor (DAC or RG108). Data represent methylation frequencies from two independent experiments. (B) Expression levels of *CYP* genes were examined by real-time RT-PCR in hESC-Hep treated with DMSO, DAC, or RG108. Data represent mean ± SD from three independent experiments. * p<0.05, ** p<0.01, significant values in comparison with DMSO (ANOVA followed by Dunn’s multiple comparison test). (C) Expression levels of *CYP* genes were examined by real-time RT-PCR in hESC-Hep treated with DMSO or a HDAC inhibitor (1 mM sodium butyrate (SB)) with or without a DNMT inhibitor (DAC or RG108). Data represent mean ± SD from three independent experiments. * p<0.05, ** p<0.01, *** p<0.001, significant values in comparison with DMSO (ANOVA followed by Bonferroni’s multiple comparison test).

We validated the transcript levels of epigenetic-related genes such as DNA methyltransferases (DNMTs), histone deacetylases (HDACs), and sirtuins (SIRTs) in hPH and hESC-Hep. *DNMT3B*, *HDAC1*, *HDAC2*, and *HDAC3* were highly expressed in hESC-Hep compared to hPH (S4A and S4B Fig). However, expression of *SIRT1* and *SIRT3* genes was lower in hESC-Hep than in hPH (S4C Fig). These results represent that epigenetic regulatory mechanism of hESC-Hep differs from that of hPH. To investigate whether DNA methylation directly modulates the expression of these five *CYP* genes in hESC-Hep, we treated cells with a DNMT inhibitor including decitabine (DAC, 2’-deoxy-5-azacytidine) and RG108 during hepatic maturation and examined changes in DNA methylation and expression of *CYP* genes. Methylation frequencies in the *CYP1A1* and *CYP1B1* promoter regions were lower in DAC- and RG108-treated hESC-Hep than in vehicle-treated hESC-Hep (Fig 4A). *CYP1A1* was up-regulated at the transcript level in DAC- and RG108-treated cells, whereas expression of *CYP1B1* was not affected (Fig 4B). In *CYP1A2*, transcript level was increased in DAC- and RG108-treated cells, although DNA methylation in two regulatory regions was not significantly changed (Fig 4A and 4B). DNMT inhibition did not seem to affect the methylation frequencies at regulatory regions and transcript levels of *CYP2D6* and *CYP2E1* compared to DMSO-treated cells (Fig 4A and 4B). However, expression level of *CYP2E1* was different between DAC- and RG108-treated cells (Fig 4A). We also investigated transcriptional regulation by DNMT inhibition in hepatocytes derived from human induced pluripotent stem cells (hiPSCs, S5 Fig). hiPSCs were also efficiently differentiated into hepatocytes, which expressed hepatocyte markers and had liver functions including glycogen storage, LDL uptake, secretion of albumin, and synthesis of urea (S6 Fig). Transcriptional activation in *CYP1A1* and *CYP1A2* was found in DAC- and RG108-treated hiPSC-Hep, whereas *CYP1B1*, *CYP2D6*, and *CYP2E1* were not (S7A Fig). These results indicate that DNA methylation is involved in transcriptional regulation of *CYP1A1* and *CYP1A2* during the differentiation of hESCs into hepatocytes.

Next, we tested the combined effect of DNMT and HDAC inhibition on the expression of CYP genes in hESC-Hep and hiPSC-Hep. Treatment of sodium butyrate (SB, a HDAC inhibitor) with or without DNMT inhibitors leads to significant increase in transcript levels of *CYP1A1* and *CYP1B1* in hESC-Hep (Fig 4C). Transcript levels of *CYP1A2* and *CYP2D6* were increased by co-treatment with SB/DAC and SB/DAC or RG108, respectively (Fig 4C). However, expression of *CYP2E1* after co-treatment with HDAC and DNMT inhibitors was similar to DMSO control (Fig 4C). We also found that transcriptional regulation of CYP genes by combined DNMT and HDAC inhibition in hiPSC-Hep was consistent with that in hESC-Hep (S7B Fig). Moreover, expression of *CYP1A1*, *CYP1A2*, and *CYP1B1* in hESC-Hep were also regulated by treatment with valproic acid (VPA) with or without DNMT inhibitors (S7C Fig). On the other hand, inhibition of DNMTs and HDACs might not affect the overall impact of hepatocyte differentiation efficiency, although expression of hepatocyte marker genes including *ALB* and *AAT* was slightly increased in hPSC-Heps treated with DNMT and HDAC inhibitors (S8 Fig). Taken together, these results show that transcription of *CYP* genes in hESC-Hep and hiPSC-Hep is influenced by epigenetic modulation via inhibition of DNMTs and HDACs.

## Discussion

Hepatocytes derived from human PSCs are valuable tools for drug discovery and hepatotoxicity testing. However, DME gene expression was limited in hESC-Hep (Fig 2). hESC-Hep seem to be more similar to fetal liver than to adult liver because they express AFP (Fig 1A and S2B Fig) and the most important DME genes are inactive prior to birth [17,18]. Among DME genes, CYP enzymes play a role in the biotransformation of most drugs [25] and their expression is potentially regulated by epigenetic modifications [19,21,22]. Our study detected low constitutive expression of most *CYP* genes in hESC-Hep and investigated epigenetic marks, namely, DNA methylation and histone modifications, at the regulatory regions of *CYP* genes for the first time. The epigenetic regulation of five *CYP* genes, namely, *CYP1A1*, *CYP1A2*, *CYP1B1*, *CYP2D6*, and *CYP2E1*, differed between hESC-Hep and hPH. Moreover, inhibition of DNMTs and HDACs may be involved in the regulation of *CYP* genes during the differentiation of hESCs into hepatocytes.

CYP1 family genes, including *CYP1A1*, *CYP1A2*, and *CYP1B1*, are induced by aryl hydrocarbon receptor (AHR) signaling in response to polycyclic aromatic hydrocarbon ligands [26–28]. The *CYP1A1* and *CYP1A2* genes are oriented head-to-head and share a bidirectional regulatory region [29,30]. Unexpectedly, among CYP1 family genes, constitutive expression of *CYP1A2* was extremely low in hESC-Hep (Fig 2B). Therefore, our study compared the epigenetic regulations among CYP1 family genes in hPH and hESC-Hep. The transcript levels of genes encoding major CYP enzymes, such as *CYP3A4*, *CYP2D6*, *CYP2C9*, *CYP2C19*, and *CYP2E1*, were much lower in hECS-Hep than in hPH (Fig 2B). *CYP3A4* and *CYP2C9*, which are most abundant and functional in the liver, can be induced by pregnane X receptor (PXR) and constitutive androstane receptor (CAR) [25]. Transcriptional inactivation of *CYP3A4* and *CYP2C9* in hESC-Hep would be influenced by silencing of the *NR1I2* and *NRI13* encoding PXR and CAR, respectively (Fig 2A and 2B). Also, interindividual variation of *CYP3A4* expression in fetal and adult human liver is associated with methylation of single CpGs in promoter region, which is approximately 12 kb upstream of TSS [31]. However, the detailed transcriptional regulation of *CYP2D6* and *CYP2E1* has not been extensively studied. Hence, we focused on the epigenetic regulation of members of the CYP1 family, *CYP2D6*, and *CYP2E1* during hepatocyte differentiation.

*CYP1A1* is expressed early in embryogenesis, and *CYP1A1* and *CYP1B1* are primarily expressed in extrahepatic tissues [32–34]. By contrast, *CYP1A2* is constitutively expressed at high levels only in adult liver [35]. Histone deacetylation and DNA methylation influence the constitutive expression of *CYP1A1*, *CYP1A2*, and *CYP1B1* in a cell type-specific manner [36,37]. In hPH and hESC-Hep, epigenetic modifications involved in transcriptional activation was observed in regulatory regions of *CYP1A1* and *CYP1B1*, although these cells represented different methylation frequency of CpGs and enrichment of H3K4me3 (Fig 3 and S3 Fig). Regulatory regions of *CYP1A1* and *CYP1B1* include xenobiotic response element (XRE), which is binding sites for the AHR complex [38,39]. Transcriptional activation of *CYP1A1* in hESC-Hep is influenced by unmethylated CpG sites into regulatory region, which contains two XRE [39]. Expression of *CYP1A1* and *CYP1B1* was up-regulated in hESC-Hep by epigenetic regulation via inhibition of DNMTs and HDACs (Fig 4). The methylation status of a CpG island in the second exon of *CYP1A2*, which contains 17 CpG dinucleotides, is correlated with the hepatic transcript level of this gene [40]. The low expression of *CYP1A2* in hESC-Hep was associated with the hypermethylation of CpG sites and H3K27me3 in the second exon and expression of this gene was up-regulated by inhibition of DNMTs and HDACs (Figs 2B, 3B, and 4). However, this up-regulation was not sufficient to induce enzyme activity comparable with that in hPH because CpG sites in the second exon were incompletely demethylated. Moreover, induction of *CYP1A2* gene by omeprazole and endogenous AHR agonist (ITE, 2-(1*H*-Indol-3-ylcarbonyl)-4-thiazolecarboxylic acid methyl ester) did not be altered in the presence of DNMT inhibitors during hepatocyte differentiation (S9 Fig). It seems to be correlated with enrichment of repressive histone mark H3K27me3 in hESC-Hep (Fig 3B). Polycomb group protein EZH2, which is a methyltransferase and contributes methylation of H3K27, interacts with DNMTs and is associated with DNMT activity [41,42].

*CYP2D6* and *CYP2E1* are poorly expressed in fetal liver but their expression rapidly increases within hours after birth [43–46]. CYP2D6 is associated with the metabolism of more than 50 clinically important drugs [47]. Genetic variations in *CYP2D6* have been extensively studied and well characterized, which could have special relevance for revealing *CYP2D6* interindividual variations [48]. The present study revealed that hypermethylation of CpG islands in the promoter and gene body regions of *CYP2D6* may be crucial for the down-regulation of this gene in hESC-Hep (Fig 3D). Incomplete demethylation after DNMT inhibition is likely to be associated with enrichment of H3K27me3 in regulatory region like *CYP1A2*. The transcript level of *CYP2D6* was increased after combinatorial inhibition of DNMTs and HDACs (Fig 4C). Methylation of specific 5’ residues of *CYP2E1* may be responsible for the lack of expression of this gene in fetal liver [49]. Transcription of *CYP2E1* in human lung, kidney, and full-term placenta is regulated by extensive methylation of the first exon and first intron of this gene [50]. Similarly, silencing of *CYP2E1* in hESC-Hep was associated with hypermethylation of the CpG island in the first intron (Fig 3E). This regulatory region was occupied by the active histone marks H3Ac and H3K4me3 in hPH, thereby correlating with activation of *CYP2E1* (Fig 3E). However, expression of *CYP2E1* after DNMT or DNMT/HDAC inhibition did not seem to affect or down-regulate in hESC-Hep and hiPSC-Hep (Fig 4 and S7 Fig). These results suggest that epigenetic modifications, such as DNA methylation and histone modifications, may be closely correlated with the limited transcription of *CYP* genes, including *CYP1A2*, *CYP2D6*, and *CYP2E1*, in hESC-Hep.

In summary, hESC-Hep have a limited drug metabolism ability, which restricts their use for *in vitro* hepatotoxicity testing. This is because the majority of CYP genes involved in drug metabolism, including *CYP1A2*, *CYP2C9*, *CYP2C19*, *CYP3A4*, *CYP2D6*, and *CYP2E1*, are lowly expressed in hECS-Hep. DNA methylation and histone modifications of regulatory regions of *CYP* genes differed between hPH and hESC-Hep. These differences were associated with inhibitory regulation of *CYP* genes in hESC-Hep. Inhibition of DNMTs and HDACs increased the transcription of *CYP* genes in hESC-Hep, but these increased transcripts were not comparable with that in hPH. Further studies are required to improve the expression and activity of CYP enzymes by epigenetic regulations. These findings show that expression of *CYP* genes is modulated by controlling epigenetic modification enzymes, such as DNMTs and HDACs.

## Materials and Methods

### Ethics statement

All experiments involving hESCs were approved by the ethics committee of Korea Institute of Toxicology (approval number: 2013–001) and approval of this research was reported to Korea Centers for Disease Control and Prevention. CHA-hES15 cell line was received from CHA Stem Cell Institute, CHA University, Korea.

### Differentiation of hESCs into hepatocytes

hESCs (CHA-hES15 cell line) were maintained as previously described [51]. hESCs were differentiated into hepatocytes as previously described [52] with some modifications. Briefly, hESCs were cultured for 3 days on Matrigel (Corning Life Science, Tewksbury, MA, USA) in mTeSR1 (Stem Cell Technologies, Vancouver, Canada). Thereafter, hESCs were incubated in RPMI-1640 (Lonza, Baltimore, MD, USA) containing 0.5 mg/ml bovine serum albumin (BSA, Sigma-Aldrich, St. Louis, MO, USA), 1× B27 (Invitrogen, Carlsbad, CA, USA), 50 ng/ml Activin A (Peprotech, Rocky Hill, NJ, USA), and 0.5 mM sodium butyrate (Sigma-Aldrich) for 1 day, and then further cultured for 4 days in the same medium except that the concentration of sodium butyrate was reduced to 0.1 mM. After treatment with Activin A, differentiated cells were cultured in RPMI-1640 containing 0.5 mg/ml BSA, 1× B27, 30 ng/ml fibroblast growth factor 4 (FGF4, Peprotech), and 20 ng/ml bone morphogenetic protein 2 (BMP2, Peprotech) for 5 days, and then further cultured in hepatocyte culture medium (HCM, Lonza) supplemented with 20 ng/ml hepatocyte growth factor (HGF, Peprotech) for 5 days. Hepatic maturation was induced by culturing cells in HCM supplemented with 10 ng/ml oncostatin M (Peprotech) and 0.1 μM dexamethasone (Sigma-Aldrich) for 5 days. The culture media was changed daily. After hepatic induction by treatment with FGF4 and BMP2, HCM was supplemented with DNMT inhibitor (5 μM decitabine or RG108, R&D Systems, Minneapolis, MN, USA) and/or HDAC inhibitor (1 mM sodium butyrate, Sigma-Aldrich) and HGF, followed by oncostatin M and dexamethasone. Cells treated with 0.1% DMSO (Sigma-Aldrich) were used as a negative control.

### Culture of hPH

BD Gentest Cryo Human Hepatocytes (BD Biosciences, Donor No. HFC 476) were plated in BD hepatocyte culture media according to the manufacturer’s instructions, and experiments were performed 24 hours later.

### Characterization of hESC-Hep

#### Fluorescence-activated cell sorting (FACS)

Cells were dissociated by incubation with 0.05% collagenase IV (Invitrogen) at 37°C for 15 minutes followed by incubation with Accutase (Innovative Cell Technologies, San Diego, CA, USA) at 37°C for 15 minutes. Dissociated cells were fixed and permeabilized with Foxp3 Fixation/Permeabilization solution (eBioscience, San Diego, CA, USA) for 1 hour at room temperature (RT). One microgram of mouse anti-ALB (R&D Systems) and rabbit anti-AAT (Abcam, Cambridge, UK) was conjugated using the Zenon R-Phycoerythrin Mouse IgG2a Labeling Kit and the Zenon Alexa Fluor 488 Rabbit IgG Labeling Kit (Invitrogen), respectively, according to the manufacturer’s instructions. Cells were incubated at RT for 1 hour with each labeled antibody. Cells were also labeled with the isotype control as a negative control. Flow cytometry was performed using BD FACS Calibur (BD Biosciences).

#### Immunofluorescence

Cells were fixed in 4% formaldehyde (Sigma-Aldrich) for 30 minutes at RT, rinsed three times in PBS containing 0.1% Tween 20 (PBST) for 10 minutes, permeabilized in PBS containing 0.1% Triton X-100 (Sigma-Aldrich) for 15 minutes, and blocked for 1 hour in PBS containing 5% normal goat serum (Jackson ImmunoResearch, West Grove, PA, USA). Cells were incubated overnight at 4°C with the following primary antibodies diluted in PBS containing 5% normal goat serum: rabbit anti-ALB (1:50; Dako, Glostrup, Denmark), rabbit anti-AFP (1:200; Dako); rabbit anti-AAT (1:200; Abcam), mouse anti-HNF4A (1:200; Abcam). Cells were rinsed six times in PBST for 10 minutes each. Thereafter, cells were incubated for 1 hour at RT with appropriate secondary antibodies diluted in PBST: Alexa Fluor 488 or 594 goat anti-rabbit IgG and Alexa Fluor 594 goat anti-mouse IgG (1:200; Invitrogen). Cells were washed six times in PBST, and mounted in 4'-6-diamidino-2-phenylindole (DAPI, Sigma-Aldrich).

#### Periodic acid-Schiff (PAS) staining of stored glycogen

Cells were fixed in 4% formaldehyde for 30 minutes, rinsed three times in PBST for 10 minutes, permeabilized with PBS containing 0.1% Triton X-100 for 15 minutes, and rinsed three times in PBST. Samples were stained using a PAS staining system (Sigma-Aldrich) according to the manufacturer’s instructions and observed under white light using an inverted microscope.

#### Acetylated-low-density lipoprotein (Ac-LDL) uptake

Cells were incubated with 10 μg/ml 1,1’-dioctadecyl-3,3,3’,3’-tetramethylindocarbocyanine-labeled Ac-LDL (Life Technologies, Carlsbad, CA, USA) for 5 hours. Red fluorescence was visualized by fluorescence microscopy.

#### Enzyme-linked immunosorbent assay (ELISA) of Albumin secretion

Conditioned medium was collected 24 hours after fresh medium was added and the amount of secreted albumin was measured using a Human Albumin ELISA Quantitation Kit (Bethyl Laboratory, Montgomery, TX, USA) on a Model 680 microplate reader (Bio-Rad, Hercules, CA, USA). The mean amount of secreted ALB was measured using 100 μl of conditioned medium from two culture dishes and calculated according to each standard followed by normalization to the protein content. Protein concentration was determined using a Bio-Rad Protein Assay (Bio-Rad).

#### Urea production

Conditioned medium was collected 24 hours after fresh medium was added and the amount of secreted urea was analyzed. Urea measurement kits were purchased from BioAssay Systems (Hayward, CA, USA). The experiment was performed according to the manufacturer’s instructions. The amount of secreted urea was calculated according to each standard followed by normalization to the protein content. Protein concentration was determined using a Bio-Rad Protein Assay (Bio-Rad).

#### Bile canalicular transport

Cells were washed three times with PBS and incubated with hepatocyte culture medium containing 5 μM 5-(and-6)-carboxy-2’,7’-dichlorofluorescein diacetate (Life Technologies) for 15 minutes. Cells were washed three times and observed by fluorescence microscopy.

### Real-time reverse transcription PCR (RT-PCR)

Total RNA was isolated from cells using TRIzol Reagent (Invitrogen) and reverse-transcribed using SuperScript II Reverse Transcriptase (Invitrogen) according to the manufacturer’s protocol. Gene expression levels were measured by real-time RT-PCR using Power SYBR Green PCR Master Mix (Applied Biosystems, Foster City, CA, USA). Relative expression levels were analyzed using a StepOnePlus Real-Time PCR System (Applied Biosystems) according to the manufacturer’s instructions. Triplicate PCR reactions were performed for each sample. The primers used for gene expression analysis are listed in S1 Table. For comparative quantification, results from real-time PCR were expressed as a relative fold change compared to control cells, after normalization against glyceraldehyde-3-phosphate dehydrogenase (*GAPDH*). The ΔCt (SΔCt) value was calculated as the difference between the Ct values of *GAPDH* and the target. The ΔCt value of control cells was used as the control ΔCt (CΔCt) value. Relative gene expression level was determined using the formula, 2^-(SΔCt−CΔCt)^.

### Epigenetic analysis

#### Bisulfite sequencing

Genomic DNA was isolated from cells using G-DEX IIc Genomic DNA Extraction Kit (iNtRON Biotechnology, Gyeonggi-do, Korea) according to the manufacturer’s protocol. Bisulfite conversion was performed using the EZ DNA Methylation—Gold Kit (ZYMO RESEARCH, Orange, CA, USA) according to the manufacturer’s protocol. Bisulfite-specific PCR reactions were carried out on a GeneAmp PCR System 9700 (Applied Biosystems) using the following protocol: 95°C for 15 minutes, 50 cycles of 95xC for 20 seconds, 55°C for 40 seconds, 72°C for 30 seconds, and extension at 72°C for 10 minutes. The primer sequences used for PCR are listed in S2 Table. PCR products were purified using the MEGAquick-spin Total Fragment DNA Purification Kit (iNtRON Biotechnology), cloned into pGEM T vector (Promega, Madison, WI, USA), and sequenced using an ABI 3730XL Capillary DNA sequencer (Applied Biosystems). Methylated or unmethylated states of CpG sites were determined from the sequence data by using QUMA (QUantification tool for Methylation Analysis) software [53].

#### Chromatin Immunoprecipitation

Briefly, approximately 1 × 10^6^ cells were incubated in cell culture medium containing 1.0% formaldehyde at 25°C for 10 min, and quenched by the addition of 0.125 M glycine for 5 min at 25°C. Cells were harvested by scraping, washed twice in PBS, and three times in ChIP lysis buffer, and resuspended in 200 μl of ChIP lysis buffer containing high salt. Cross-linked chromatin was fragmented by sonication, and pre-cleared with protein A/G PLUS-agarose (Santa Cruz Biotechnology, Dallas, TX, USA) at 4°C for 1 h. Each primary antibody was incubated overnight with chromatin at 4°C. Antibodies (Millipore-Upstate, Temecula, CA, USA) used for the ChIP assay were as follows: normal rabbit IgG (#12–370), anti-acetyl-Histone H3 (#06–599), anti-trimethyl-Histone H3 Lys4 (#07–473), anti-trimethyl-Histone H3 Lys27 (#07–449). Immunocomplexes were harvested by incubation with protein A/G PLUS-agarose for 2 h at 4°C. Immunoprecipitates were washed twice with lysis buffer containing high salt, and rinsed four times with wash buffer. Samples were resuspended in elution buffer and incubated at 67°C overnight. DNA samples were isolated using phenol/chloroform extraction, precipitated with ethanol, and resuspended in 50 μl of TE buffer. Quantitative PCR was carried out on a StepOnePlus Real-Time PCR System (Applied Biosystems) according to the manufacturer’s instructions. Triplicate PCR reactions were performed for each sample. The primer sequences used for PCR are listed in S3 Table. ChIP-quantitative PCR results were calculated using the ΔΔCt method. The Ct value of the respective ChIP fraction was normalized against the Ct value of the input DNA fraction (ΔCt). Then, the Ct value of the ChIP fraction was again normalized to the Ct value of the IgG control (ΔΔCt). Fold enrichment of immunoprecipitation was calculated by 2^-ΔΔCt^.

### Statistical analysis

Data obtained from two or three separate experiments were expressed as mean ± standard deviation (SD), and statistically analyzed by t-test and one-way analysis of variance (ANOVA) using GraphPad Prism software (San Diego, CA, USA). p value lower than 0.05 were considered significant.

## Supporting Information

S1 Fig Methodology used to differentiate human pluripotent stem cells (hPSCs) into hepatocytes. hPSC, human pluripotent stem cell; FGF4, fibroblast growth factor 4; BMP2, bone morphogenetic protein 2; HGF, hepatocyte growth factor; OSM, oncostatin M; Dex, dexamethasone; BSA, bovine serum albumin; SB, sodium butyrate; HCM, hepatocyte culture medium.
(TIFF)

S2 Fig Expression of definitive endoderm and hepatocyte markers. (A) FACS analysis of CXCR4-positive cells was performed 5 days after the onset of differentiation. Blue line: isotype control, red line: primary antibody. (B) Immunofluorescence labeling of albumin (ALB), α-1-antitrypsin (AAT), α-fetoprotein (AFP), and hepatocyte nuclear factor 4 α (HNF4A) was performed at day 20 of differentiation. The scale bar represents 100 μm.
(TIFF)

S3 Fig Histone modifications in regulatory regions of *CYP1A1* and *CYP1B1* genes. Each diagram shows the locations of the sites of *CYP1A1* (A) and *CYP1B1* (B) within gene promoters, which were examined by ChIP. ChIP analysis of histone modifications in hPH and hESC-Hep (day 20 of differentiation) is shown in lower graphs. Data validated by real-time PCR are presented as fold enrichment of precipitated DNA associated with a given histone modification relative to a 100-fold dilution of input chromatin. Data represent mean ± SD from two independent experiments. * p<0.05, significant values in comparison with hPH (t-test followed by Wilcoxon matched pairs test).
(TIFF)

S4 Fig Gene expression levels of epigenetic modification enzymes. Expression of genes encoding DNMTs (A), HDACs (B), and Sirtuins (C) was examined by real-time RT-PCR in hPH and hESC-Hep (day 20). Data represent mean ± SD from three independent experiments. * p<0.05, significant values in comparison with hPH (t-test followed by Wilcoxon matched pairs test).
(TIFF)

S5 Fig Characterization of human induced pluripotent stem cells (hiPSCs). (A) Immunofluorescence detection of pluirpotency markers including OCT4, SOX2, SSEA4, and TRA-1-60 in hiPSCs was performed at after 4 days culture on feeder cells. Insets show DAPI staining. Scale bar, 100 μm. (B) RT-PCR analysis of endogenous pluripotency marker genes including *OCT4*, *SOX2*, *CMYC*, *KLF4*, *REX1*, *ECAD*, and *TERT* was examined in hESCs (CHA-hES15), fibroblasts, and hiPSCs. (C) DNA methylation on promoters of pluripotency marker genes including *OCT4*, *REX1*, and *NANOG* was performed by bisulfite sequencing in fibroblasts and hiPSCs. Each row of circles represents the methylation status of each CpG in one bacterial clone. Open and filled circles indicate unmethylated and methylated CpG dinucleotides, respectively. (D) G-banded karyotyping analysis of hiPSCs was performed at passage 31. (E) Teratoma formation of hiPSCs in immunodeficient mice. Hematoxylin and eosin (H&E) staining was performed on formalin-fixed teratoma sections showing ectoderm (a, neural tissue), mesoderm (b, smooth muscle and adipocyte) and endoderm (c, gut) tissues.
(TIFF)

S6 Fig Differentiation of hiPSCs into hepatocytes. (A) Immunofluorescence labeling of ALB, AAT, AFP, and HNF4A was performed at day 20 of differentiation. The scale bar represents 100 μm. (B) FACS analysis of ALB-positive cells was performed 20 days after the onset of differentiation. Blue line: isotype control, red line: primary antibody. (C) Glycogen storage and Ac-LDL uptake in hiPSC-Hep. Periodic acid-Schiff staining of glycogen was performed at day 20 of differentiation. Stored glycogen (purple) was observed in the cytoplasm. Nuclei (light blue) were counterstained with hematoxylin. The ability of cells to take up Ac-LDL was examined at day 20 of differentiation. The scale bar represents 100 μm. (D) ALB secretion from hiPSC-Hep. The ALB concentration was measured in the conditioned media of hiPSCs (day 0), hiPSC-Hep (day 20), and hPH by an enzyme-linked immunosorbent assay using an anti-human ALB antibody. (E) Urea production by hiPSC-Hep. The amount of urea secreted by hiPSCs (day 0), hiPSC-Hep (day 20), and hPH was examined at 0, 24, and 48 hours.
(TIFF)

S7 Fig Transcriptional regulation of *CYP* genes by inhibition of DNMTs and HDACs during differentiation of hPSCs into hepatocytes. (A) Expression levels of *CYP* genes were examined by real-time RT-PCR in hiPSC-Hep treated with DMSO, DAC, or RG108. Data represent mean ± SD. (B) Expression levels of *CYP* genes were examined by real-time RT-PCR in hiPSC-Hep treated with DMSO or SB with or without DAC or RG108. Data represent mean ± SD. (C) Expression levels of *CYP* genes were examined by real-time RT-PCR in hESC-Hep treated with DMSO or 2 mM valproic acid (VPA) with or without a DNMT inhibitor (DAC or RG108). Data represent mean ± SD.
(TIFF)

S8 Fig Expression of hepatocyte marker genes by inhibition of DNMTs and HDACs during differentiation of hPSCs into hepatocytes. (A and B) Expression levels of *ALB* (A) and *AAT* (B) were examined by real-time RT-PCR in hPSC-Heps treated with DMSO, DAC, RG108, SB, or SB with DAC or RG108. Data represent mean ± SD. (C) Percentages of ALB and AAT positive cells was performed by FACS analysis in hESC-Hep treated with DMSO or SB.
(TIF)

S9 Fig Transcriptional regulation of *CYP1A2* gene by CYP inducer with inhibition of DNMTs. Expression level of *CYP1A2* gene was examined by real-time RT-PCR in hESC-Hep (A) and hiPSC-Hep (B) treated with DMSO, DAC, or RG108 at day 15 of differentiation for 5 days and then further treated with 100 μM OME (omeprazole) or 0.5 μM ITE at day 19 of differentiation for 24 hr. Data represent mean ± SD.
(TIF)

S1 Table Primers used for real-time reverse transcription PCR analysis. (DOCX)

S2 Table Primers used for bisulfite sequencing. (DOCX)

S3 Table Primers used for chromatin immunoprecipitation. (DOCX)

S1 Materials and Methods Supporting Materials and Methods. (DOCX)

## References

[1] Guguen-Guillouzo C, Corlu A, Guillouzo A (2010) Stem cell-derived hepatocytes and their use in toxicology. Toxicology 270: 3–9. 10.1016/j.tox.2009.09.019 19815049

[2] Szkolnicka D, Zhou W, Lucendo-Villarin B, Hay DC (2013) Pluripotent stem cell-derived hepatocytes: potential and challenges in pharmacology. Annu Rev Pharmacol Toxicol 53: 147–159. 10.1146/annurev-pharmtox-011112-140306 23294308

[3] Scott CW, Peters MF, Dragan YP (2013) Human induced pluripotent stem cells and their use in drug discovery for toxicity testing. Toxicol Lett 219: 49–58. 10.1016/j.toxlet.2013.02.020 23470867

[4] Godoy P, Hewitt NJ, Albrecht U, Andersen ME, Ansari N, Bhattacharya S, et al (2013) Recent advances in 2D and 3D in vitro systems using primary hepatocytes, alternative hepatocyte sources and non-parenchymal liver cells and their use in investigating mechanisms of hepatotoxicity, cell signaling and ADME. Arch Toxicol 87: 1315–1530. 10.1007/s00204-013-1078-5 23974980 PMC3753504

[5] Sjogren AK, Liljevald M, Glinghammar B, Sagemark J, Li XQ, Jonebring A, et al (2014) Critical differences in toxicity mechanisms in induced pluripotent stem cell-derived hepatocytes, hepatic cell lines and primary hepatocytes. Arch Toxicol 88: 1427–1437. 10.1007/s00204-014-1265-z 24912781

[6] Holmgren G, Sjogren AK, Barragan I, Sabirsh A, Sartipy P, Synnergren J, et al (2014) Long-term chronic toxicity testing using human pluripotent stem cell-derived hepatocytes. Drug Metab Dispos 42: 1401–1406. 10.1124/dmd.114.059154 24980256

[7] Takayama K, Morisaki Y, Kuno S, Nagamoto Y, Harada K, Furukawa N, et al (2014) Prediction of interindividual differences in hepatic functions and drug sensitivity by using human iPS-derived hepatocytes. Proc Natl Acad Sci U S A 111: 16772–16777. 10.1073/pnas.1413481111 25385620 PMC4250156

[8] Hannan NR, Segeritz CP, Touboul T, Vallier L (2013) Production of hepatocyte-like cells from human pluripotent stem cells. Nat Protoc 8: 430–437. 23424751 10.1038/nprot.2012.153 PMC3673228

[9] Takayama K, Kawabata K, Nagamoto Y, Kishimoto K, Tashiro K, Sakurai F, et al (2013) 3D spheroid culture of hESC/hiPSC-derived hepatocyte-like cells for drug toxicity testing. Biomaterials 34: 1781–1789. 10.1016/j.biomaterials.2012.11.029 23228427

[10] Gieseck RL 3rd, Hannan NR, Bort R, Hanley NA, Drake RA, Cameron GW, et al (2014) Maturation of induced pluripotent stem cell derived hepatocytes by 3D-culture. PLoS One 9: e86372 10.1371/journal.pone.0086372 24466060 PMC3899231

[11] Berger DR, Ware BR, Davidson MD, Allsup SR, Khetani SR (2014) Enhancing the functional maturity of iPSC-derived human hepatocytes via controlled presentation of cell-cell interactions in vitro. Hepatology. 10.1002/hep.27621 25421237

[12] Kondo Y, Iwao T, Yoshihashi S, Mimori K, Ogihara R, Nagata K, et al (2014) Histone deacetylase inhibitor valproic acid promotes the differentiation of human induced pluripotent stem cells into hepatocyte-like cells. PLoS One 9: e104010 10.1371/journal.pone.0104010 25084468 PMC4119015

[13] Baxter M, Withey S, Harrison S, Segeritz C, Zhang F, Atkinson-Dell R, et al (2014) Phenotypic and functional analyses show stem cell-derived hepatocyte-like cells better mimic fetal rather than adult hepatocytes. J Hepatol. 10.1016/j.jhep.2014.10.016 PMC4334496 25457200

[14] Basma H, Soto-Gutierrez A, Yannam GR, Liu L, Ito R, Yamamoto T, et al (2009) Differentiation and transplantation of human embryonic stem cell-derived hepatocytes. Gastroenterology 136: 990–999. 10.1053/j.gastro.2008.10.047 19026649 PMC2732349

[15] Kia R, Sison RL, Heslop J, Kitteringham NR, Hanley N, Mills JS, et al (2013) Stem cell-derived hepatocytes as a predictive model for drug-induced liver injury: are we there yet? Br J Clin Pharmacol 75: 885–896. 10.1111/j.1365-2125.2012.04360.x 22703588 PMC3612706

[16] Hart SN, Cui Y, Klaassen CD, Zhong XB (2009) Three patterns of cytochrome P450 gene expression during liver maturation in mice. Drug Metab Dispos 37: 116–121. 10.1124/dmd.108.023812 18845660 PMC2683655

[17] Lee JS, Ward WO, Liu J, Ren H, Vallanat B, Delker D, et al (2011) Hepatic xenobiotic metabolizing enzyme and transporter gene expression through the life stages of the mouse. PLoS One 6: e24381 10.1371/journal.pone.0024381 21931700 PMC3169610

[18] Lee JS, Ward WO, Knapp G, Ren H, Vallanat B, Abbott B, et al (2012) Transcriptional ontogeny of the developing liver. BMC Genomics 13: 33 10.1186/1471-2164-13-33 22260730 PMC3306746

[19] Zhong XB, Leeder JS (2013) Epigenetic regulation of ADME-related genes: focus on drug metabolism and transport. Drug Metab Dispos 41: 1721–1724. 10.1124/dmd.113.053942 23935066 PMC3920173

[20] Bird A (2007) Perceptions of epigenetics. Nature 447: 396–398. 17522671 10.1038/nature05913

[21] Hirota T, Takane H, Higuchi S, Ieiri I (2008) Epigenetic regulation of genes encoding drug-metabolizing enzymes and transporters; DNA methylation and other mechanisms. Curr Drug Metab 9: 34–38. 18220569 10.2174/138920008783331130

[22] Kacevska M, Ivanov M, Ingelman-Sundberg M (2011) Perspectives on epigenetics and its relevance to adverse drug reactions. Clin Pharmacol Ther 89: 902–907. 10.1038/clpt.2011.21 21508940

[23] Bonder MJ, Kasela S, Kals M, Tamm R, Lokk K, Barragan I, et al (2014) Genetic and epigenetic regulation of gene expression in fetal and adult human livers. BMC Genomics 15: 860 10.1186/1471-2164-15-860 25282492 PMC4287518

[24] Weng MK, Natarajan K, Scholz D, Ivanova VN, Sachinidis A, Hengstler JG, et al (2014) Lineage-specific regulation of epigenetic modifier genes in human liver and brain. PLoS One 9: e102035 10.1371/journal.pone.0102035 25054330 PMC4108363

[25] Zanger UM, Schwab M (2013) Cytochrome P450 enzymes in drug metabolism: regulation of gene expression, enzyme activities, and impact of genetic variation. Pharmacol Ther 138: 103–141. 10.1016/j.pharmthera.2012.12.007 23333322

[26] Hankinson O (1995) The aryl hydrocarbon receptor complex. Annu Rev Pharmacol Toxicol 35: 307–340. 7598497 10.1146/annurev.pa.35.040195.001515

[27] Rowlands JC, Gustafsson JA (1997) Aryl hydrocarbon receptor-mediated signal transduction. Crit Rev Toxicol 27: 109–134. 9099515 10.3109/10408449709021615

[28] Xu C, Li CY, Kong AN (2005) Induction of phase I, II and III drug metabolism/transport by xenobiotics. Arch Pharm Res 28: 249–268. 15832810 10.1007/BF02977789

[29] Jorge-Nebert LF, Jiang Z, Chakraborty R, Watson J, Jin L, McGarvey ST, et al (2010) Analysis of human CYP1A1 and CYP1A2 genes and their shared bidirectional promoter in eight world populations. Hum Mutat 31: 27–40. 10.1002/humu.21132 19802894 PMC2797837

[30] Ueda R, Iketaki H, Nagata K, Kimura S, Gonzalez FJ, Kusano K, et al (2006) A common regulatory region functions bidirectionally in transcriptional activation of the human CYP1A1 and CYP1A2 genes. Mol Pharmacol 69: 1924–1930. 16505155 10.1124/mol.105.021220

[31] Kacevska M, Ivanov M, Wyss A, Kasela S, Milani L, Rane A, et al (2012) DNA methylation dynamics in the hepatic CYP3A4 gene promoter. Biochimie 94: 2338–2344. 10.1016/j.biochi.2012.07.013 22906825

[32] Bieche I, Narjoz C, Asselah T, Vacher S, Marcellin P, Lidereau R, et al (2007) Reverse transcriptase-PCR quantification of mRNA levels from cytochrome (CYP)1, CYP2 and CYP3 families in 22 different human tissues. Pharmacogenet Genomics 17: 731–742. 17700362 10.1097/FPC.0b013e32810f2e58

[33] Chang TK, Chen J, Pillay V, Ho JY, Bandiera SM (2003) Real-time polymerase chain reaction analysis of CYP1B1 gene expression in human liver. Toxicol Sci 71: 11–19. 12520071 10.1093/toxsci/71.1.11

[34] Stiborova M, Martinek V, Rydlova H, Koblas T, Hodek P (2005) Expression of cytochrome P450 1A1 and its contribution to oxidation of a potential human carcinogen 1-phenylazo-2-naphthol (Sudan I) in human livers. Cancer Lett 220: 145–154. 15766589 10.1016/j.canlet.2004.07.036

[35] Kawakami H, Ohtsuki S, Kamiie J, Suzuki T, Abe T, Terasaki T (2011) Simultaneous absolute quantification of 11 cytochrome P450 isoforms in human liver microsomes by liquid chromatography tandem mass spectrometry with in silico target peptide selection. J Pharm Sci 100: 341–352. 10.1002/jps.22255 20564338

[36] Beedanagari SR, Taylor RT, Bui P, Wang F, Nickerson DW, Hankinson O (2010) Role of epigenetic mechanisms in differential regulation of the dioxin-inducible human CYP1A1 and CYP1B1 genes. Mol Pharmacol 78: 608–616. 10.1124/mol.110.064899 20631054 PMC2981391

[37] Nakajima M, Iwanari M, Yokoi T (2003) Effects of histone deacetylation and DNA methylation on the constitutive and TCDD-inducible expressions of the human CYP1 family in MCF-7 and HeLa cells. Toxicol Lett 144: 247–256. 12927368 10.1016/s0378-4274(03)00216-9

[38] Tsuchiya Y, Nakajima M, Yokoi T (2003) Critical enhancer region to which AhR/ARNT and Sp1 bind in the human CYP1B1 gene. J Biochem 133: 583–592. 12801909 10.1093/jb/mvg075

[39] Tekpli X, Zienolddiny S, Skaug V, Stangeland L, Haugen A, Mollerup S (2012) DNA methylation of the CYP1A1 enhancer is associated with smoking-induced genetic alterations in human lung. Int J Cancer 131: 1509–1516. 10.1002/ijc.27421 22213191

[40] Ghotbi R, Gomez A, Milani L, Tybring G, Syvanen AC, Bertilsson L, et al (2009) Allele-specific expression and gene methylation in the control of CYP1A2 mRNA level in human livers. Pharmacogenomics J 9: 208–217. 10.1038/tpj.2009.4 19274061

[41] Vire E, Brenner C, Deplus R, Blanchon L, Fraga M, Didelot C, et al (2006) The Polycomb group protein EZH2 directly controls DNA methylation. Nature 439: 871–874. 16357870 10.1038/nature04431

[42] Hagarman JA, Motley MP, Kristjansdottir K, Soloway PD (2013) Coordinate regulation of DNA methylation and H3K27me3 in mouse embryonic stem cells. PLoS One 8: e53880 10.1371/journal.pone.0053880 23326524 PMC3543269

[43] Czekaj P, Wiaderkiewicz A, Florek E, Wiaderkiewicz R (2005) Tobacco smoke-dependent changes in cytochrome P450 1A1, 1A2, and 2E1 protein expressions in fetuses, newborns, pregnant rats, and human placenta. Arch Toxicol 79: 13–24. 15448981 10.1007/s00204-004-0607-7

[44] Rich KJ, Boobis AR (1997) Expression and inducibility of P450 enzymes during liver ontogeny. Microsc Res Tech 39: 424–435. 9408909 10.1002/(SICI)1097-0029(19971201)39:5<424::AID-JEMT5>3.0.CO;2-G

[45] Treluyer JM, Jacqz-Aigrain E, Alvarez F, Cresteil T (1991) Expression of CYP2D6 in developing human liver. Eur J Biochem 202: 583–588. 1722149 10.1111/j.1432-1033.1991.tb16411.x

[46] Vieira I, Sonnier M, Cresteil T (1996) Developmental expression of CYP2E1 in the human liver. Hypermethylation control of gene expression during the neonatal period. Eur J Biochem 238: 476–483. 8681961 10.1111/j.1432-1033.1996.0476z.x

[47] Hasler JA (1999) Pharmacogenetics of cytochromes P450. Mol Aspects Med 20: 12–24, 25–137. 10575648 10.1016/s0098-2997(99)00005-9

[48] Ingelman-Sundberg M, Sim SC, Gomez A, Rodriguez-Antona C (2007) Influence of cytochrome P450 polymorphisms on drug therapies: pharmacogenetic, pharmacoepigenetic and clinical aspects. Pharmacol Ther 116: 496–526. 18001838 10.1016/j.pharmthera.2007.09.004

[49] Jones SM, Boobis AR, Moore GE, Stanier PM (1992) Expression of CYP2E1 during human fetal development: methylation of the CYP2E1 gene in human fetal and adult liver samples. Biochem Pharmacol 43: 1876–1879. 1575782 10.1016/0006-2952(92)90726-y

[50] Vieira I, Pasanen M, Raunio H, Cresteil T (1998) Expression of CYP2E1 in human lung and kidney during development and in full-term placenta: a differential methylation of the gene is involved in the regulation process. Pharmacol Toxicol 83: 183–187. 9834965 10.1111/j.1600-0773.1998.tb01466.x

[51] Lee JE, Kang MS, Park MH, Shim SH, Yoon TK, Chung HM, et al (2010) Evaluation of 28 human embryonic stem cell lines for use as unrelated donors in stem cell therapy: implications of HLA and ABO genotypes. Cell Transplant 19: 1383–1395. 10.3727/096368910X513991 20587141

[52] Cai J, Zhao Y, Liu Y, Ye F, Song Z, Qin H, et al (2007) Directed differentiation of human embryonic stem cells into functional hepatic cells. Hepatology 45: 1229–1239. 17464996 10.1002/hep.21582

[53] Kumaki Y, Oda M, Okano M (2008) QUMA: quantification tool for methylation analysis. Nucleic Acids Res 36: W170–175. 10.1093/nar/gkn294 18487274 PMC2447804

