# Supplementary figures and images for: Differences in the Epigenetic Regulation of Cytochrome P450 Genes between Human Embryonic Stem Cell-Derived Hepatocytes and Primary Hepatocytes
Source: PLoS One. 2015 Jul 15;10(7):e0132992. doi: 10.1371/journal.pone.0132992 (PMC4503736; doi:10.1371/journal.pone.0132992)

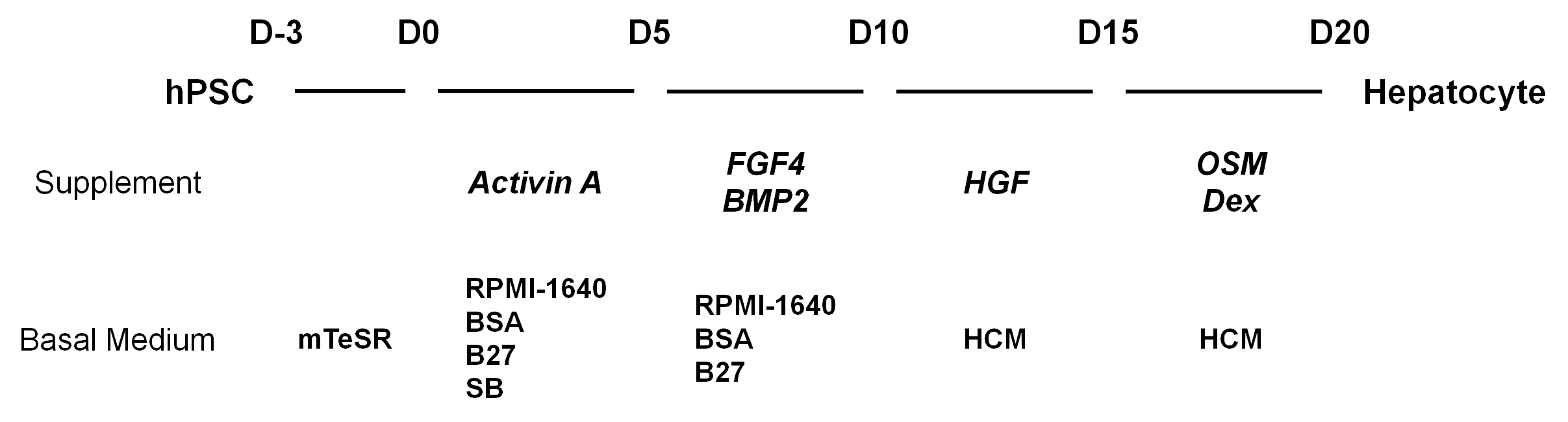

Supplement: S1 Fig — hPSC, human pluripotent stem cell; FGF4, fibroblast growth factor 4; BMP2, bone morphogenetic protein 2; HGF, hepatocyte growth factor; OSM, oncostatin M; Dex, dexamethasone; BSA, bovine serum albumin; SB, sodium butyrate; HCM, hepatocyte culture medium. (TIFF) [file pone.0132992.s001.TIFF]

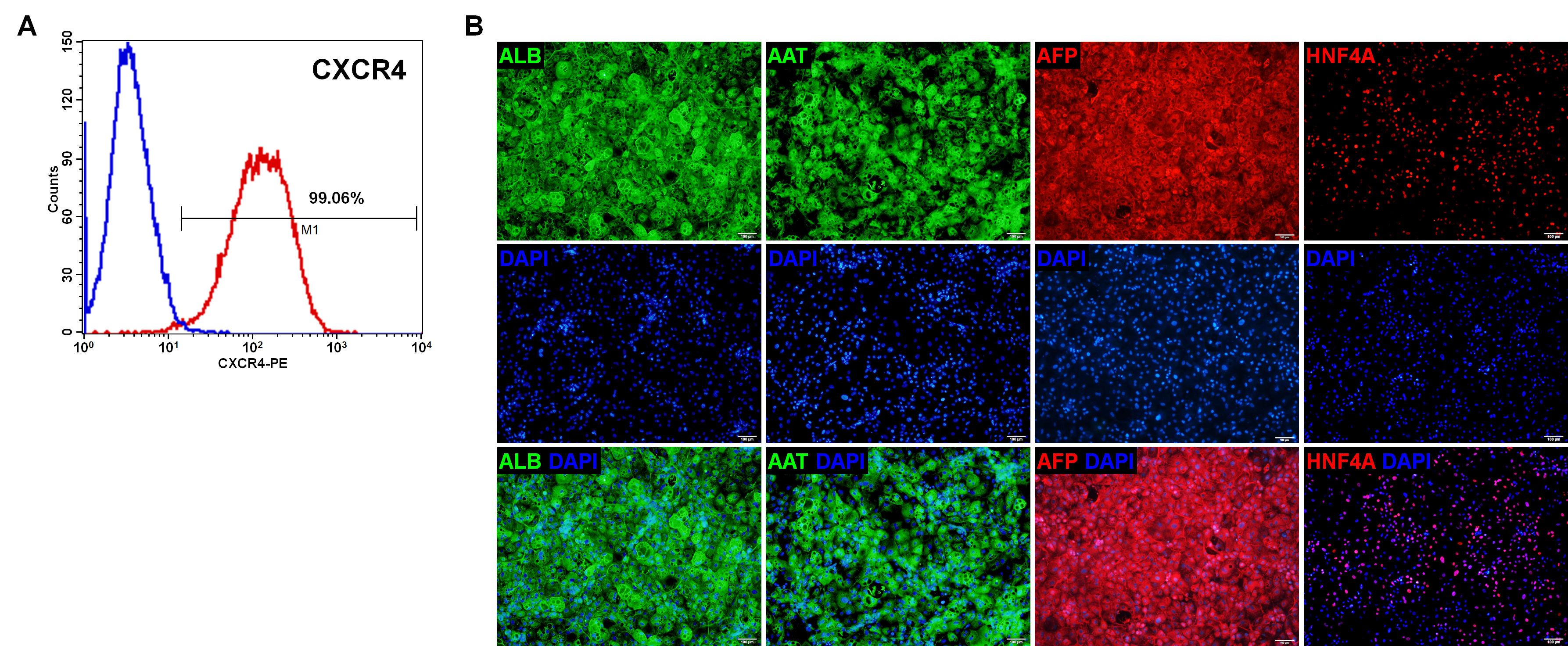

Supplement: S2 Fig — (A) FACS analysis of CXCR4-positive cells was performed 5 days after the onset of differentiation. Blue line: isotype control, red line: primary antibody. (B) Immunofluorescence labeling of albumin (ALB), α-1-antitrypsin (AAT), α-fetoprotein (AFP), and hepatocyte nuclear factor 4 α (HNF4A) was performed at day 20 of differentiation. The scale bar represents 100 μm. (TIFF) [file pone.0132992.s002.TIFF]

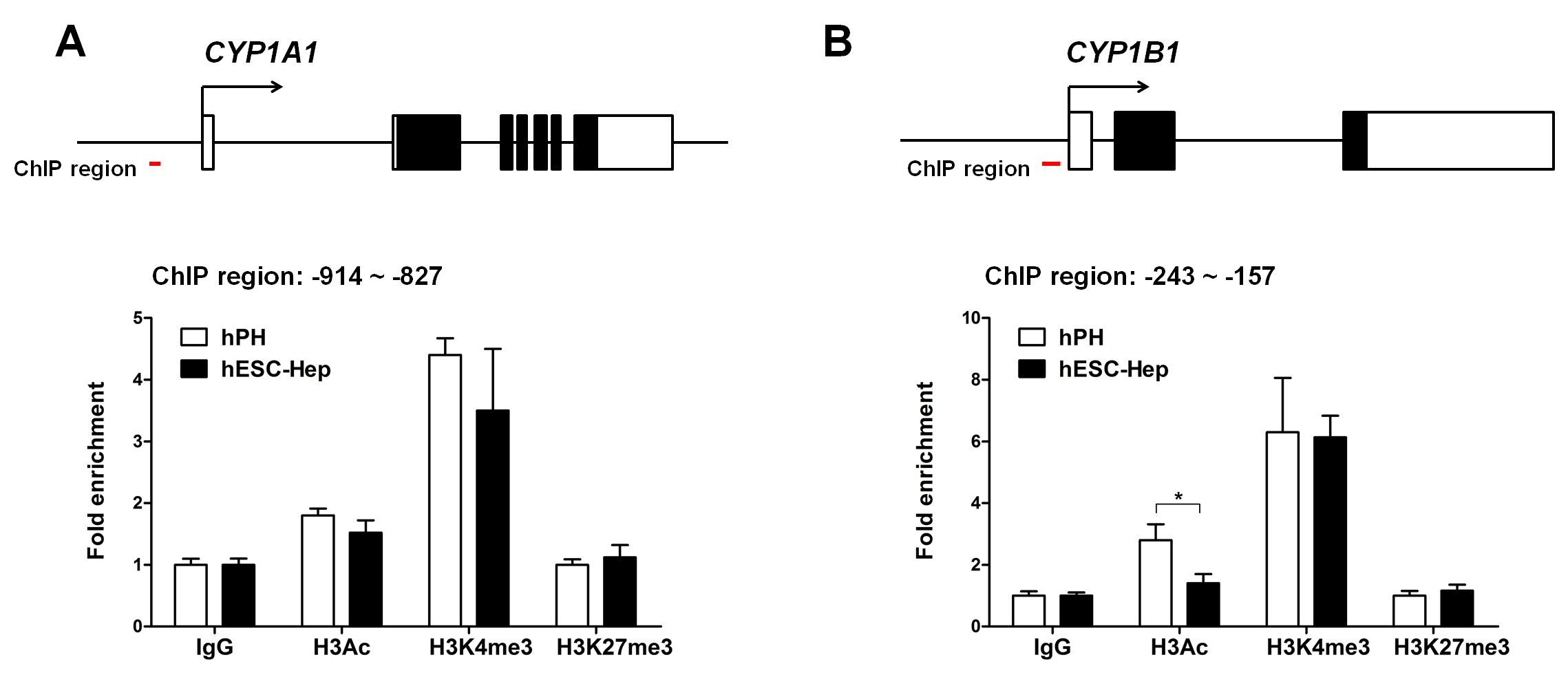

Supplement: S3 Fig — Each diagram shows the locations of the sites of CYP1A1 (A) and CYP1B1 (B) within gene promoters, which were examined by ChIP. ChIP analysis of histone modifications in hPH and hESC-Hep (day 20 of differentiation) is shown in lower graphs. Data validated by real-time PCR are presented as fold enrichment of precipitated DNA associated with a given histone modification relative to a 100-fold dilution of input chromatin. Data represent mean ± SD from two independent experiments. * p<0.05, significant values in comparison with hPH (t-test followed by Wilcoxon matched pairs test). (TIFF) [file pone.0132992.s003.TIFF]

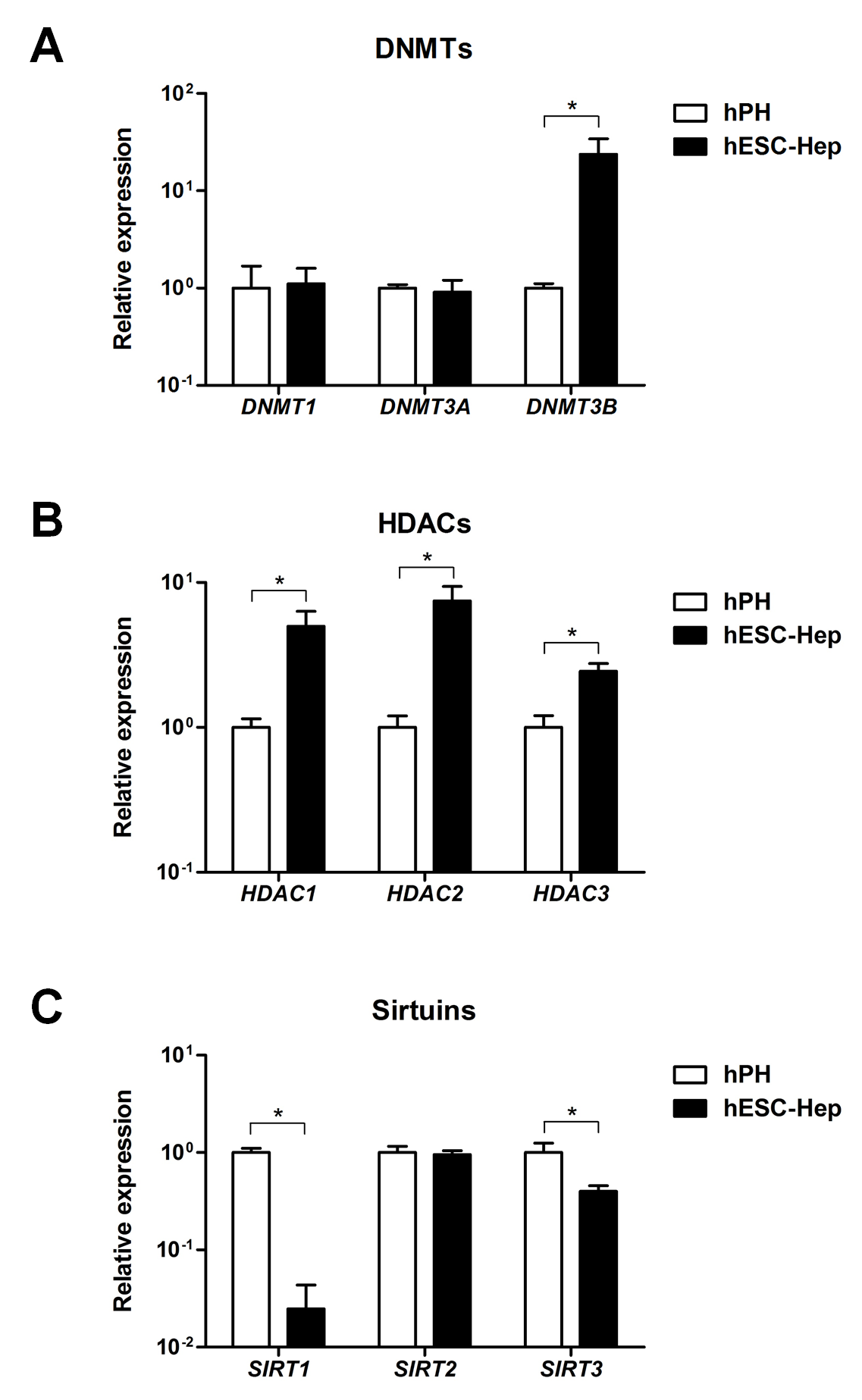

Supplement: S4 Fig — Expression of genes encoding DNMTs (A), HDACs (B), and Sirtuins (C) was examined by real-time RT-PCR in hPH and hESC-Hep (day 20). Data represent mean ± SD from three independent experiments. * p<0.05, significant values in comparison with hPH (t-test followed by Wilcoxon matched pairs test). (TIFF) [file pone.0132992.s004.TIFF]

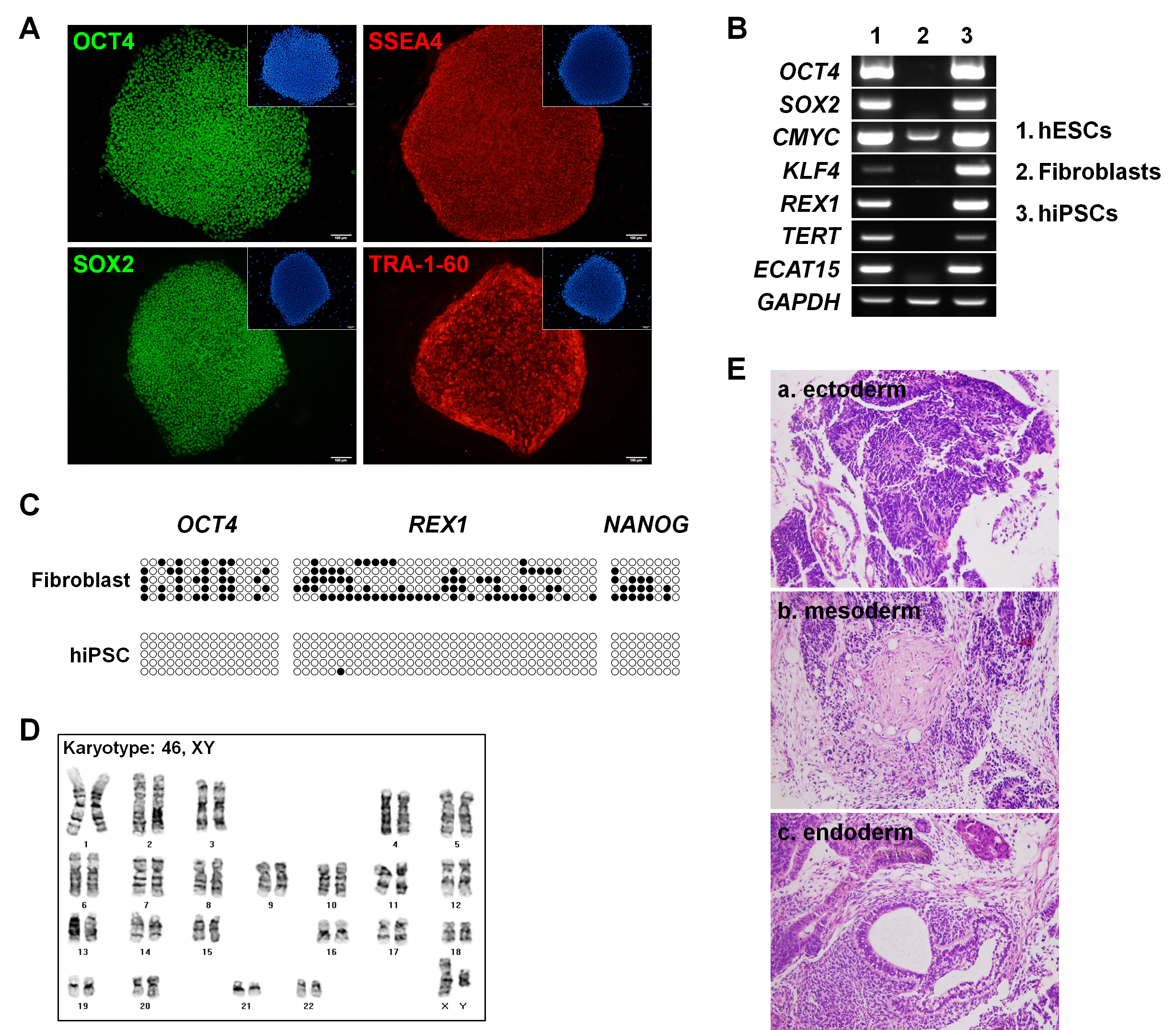

Supplement: S5 Fig — (A) Immunofluorescence detection of pluirpotency markers including OCT4, SOX2, SSEA4, and TRA-1-60 in hiPSCs was performed at after 4 days culture on feeder cells. Insets show DAPI staining. Scale bar, 100 μm. (B) RT-PCR analysis of endogenous pluripotency marker genes including OCT4, SOX2, CMYC, KLF4, REX1, ECAD, and TERT was examined in hESCs (CHA-hES15), fibroblasts, and hiPSCs. (C) DNA methylation on promoters of pluripotency marker genes including OCT4, REX1, and NANOG was performed by bisulfite sequencing in fibroblasts and hiPSCs. Each row of circles represents the methylation status of each CpG in one bacterial clone. Open and filled circles indicate unmethylated and methylated CpG dinucleotides, respectively. (D) G-banded karyotyping analysis of hiPSCs was performed at passage 31. (E) Teratoma formation of hiPSCs in immunodeficient mice. Hematoxylin and eosin (H&E) staining was performed on formalin-fixed teratoma sections showing ectoderm (a, neural tissue), mesoderm (b, smooth muscle and adipocyte) and endoderm (c, gut) tissues. (TIFF) [file pone.0132992.s005.TIFF]

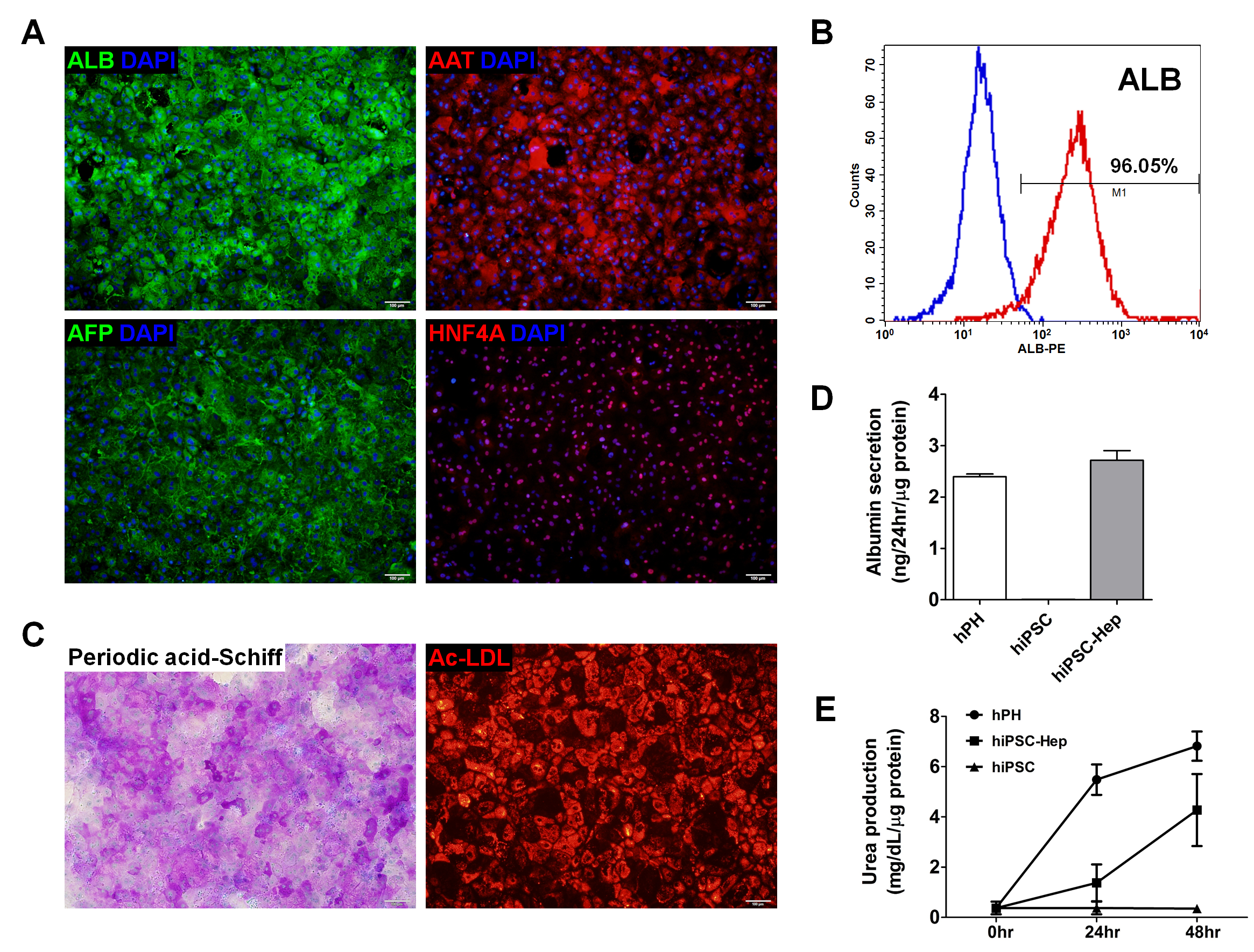

Supplement: S6 Fig — (A) Immunofluorescence labeling of ALB, AAT, AFP, and HNF4A was performed at day 20 of differentiation. The scale bar represents 100 μm. (B) FACS analysis of ALB-positive cells was performed 20 days after the onset of differentiation. Blue line: isotype control, red line: primary antibody. (C) Glycogen storage and Ac-LDL uptake in hiPSC-Hep. Periodic acid-Schiff staining of glycogen was performed at day 20 of differentiation. Stored glycogen (purple) was observed in the cytoplasm. Nuclei (light blue) were counterstained with hematoxylin. The ability of cells to take up Ac-LDL was examined at day 20 of differentiation. The scale bar represents 100 μm. (D) ALB secretion from hiPSC-Hep. The ALB concentration was measured in the conditioned media of hiPSCs (day 0), hiPSC-Hep (day 20), and hPH by an enzyme-linked immunosorbent assay using an anti-human ALB antibody. (E) Urea production by hiPSC-Hep. The amount of urea secreted by hiPSCs (day 0), hiPSC-Hep (day 20), and hPH was examined at 0, 24, and 48 hours. (TIFF) [file pone.0132992.s006.TIFF]

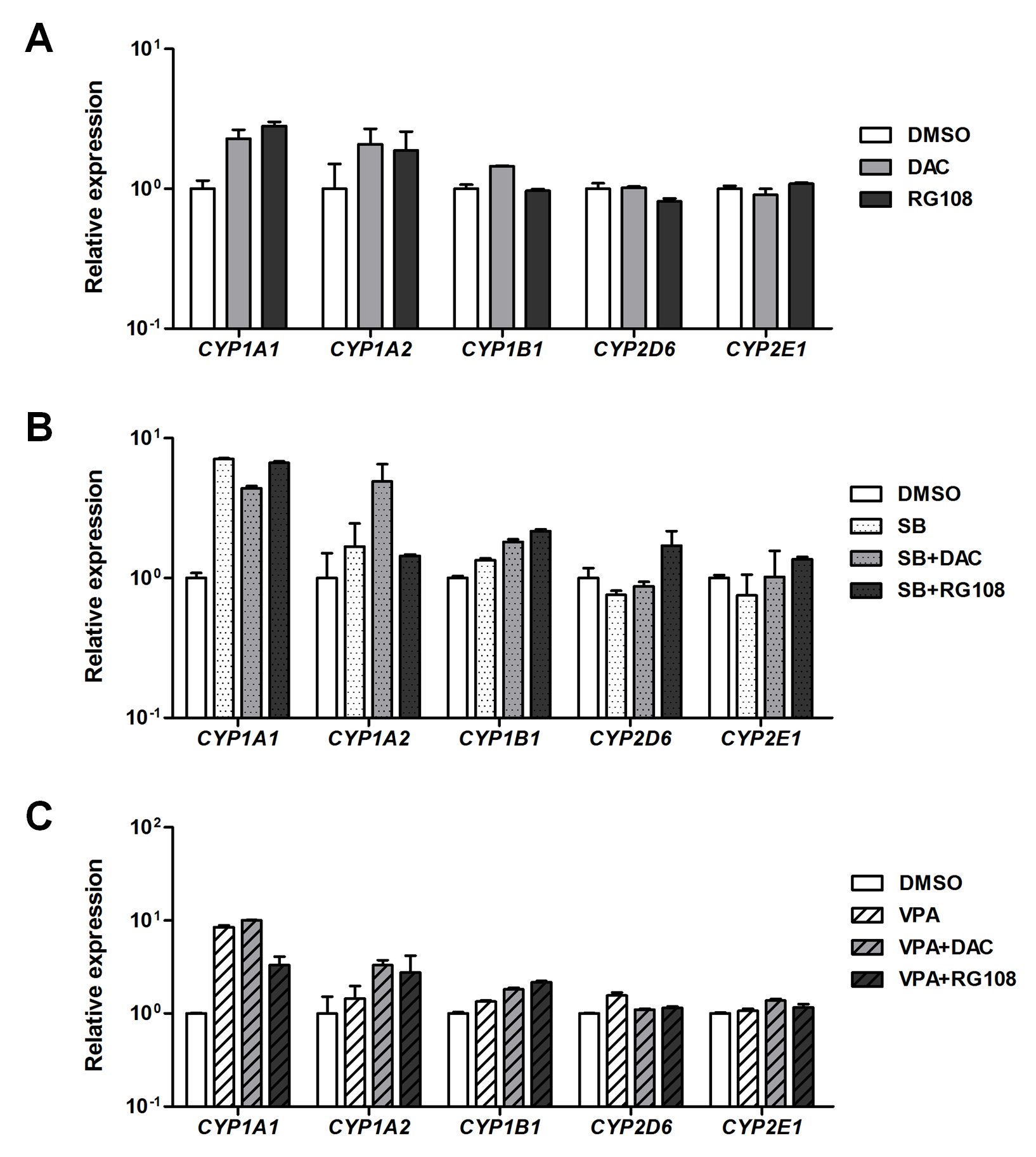

Supplement: S7 Fig — (A) Expression levels of CYP genes were examined by real-time RT-PCR in hiPSC-Hep treated with DMSO, DAC, or RG108. Data represent mean ± SD. (B) Expression levels of CYP genes were examined by real-time RT-PCR in hiPSC-Hep treated with DMSO or SB with or without DAC or RG108. Data represent mean ± SD. (C) Expression levels of CYP genes were examined by real-time RT-PCR in hESC-Hep treated with DMSO or 2 mM valproic acid (VPA) with or without a DNMT inhibitor (DAC or RG108). Data represent mean ± SD. (TIFF) [file pone.0132992.s007.TIFF]

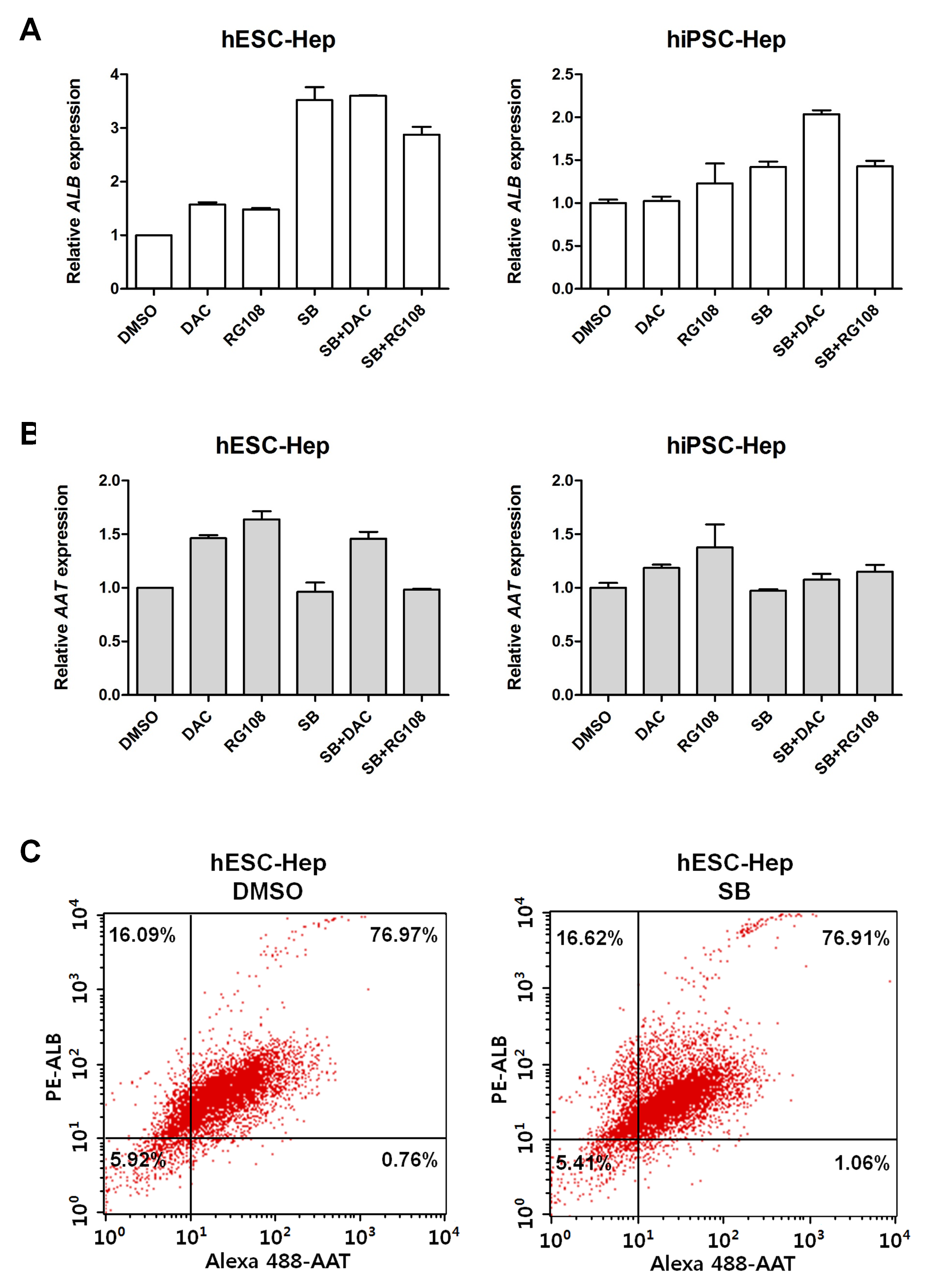

Supplement: S8 Fig — (A and B) Expression levels of ALB (A) and AAT (B) were examined by real-time RT-PCR in hPSC-Heps treated with DMSO, DAC, RG108, SB, or SB with DAC or RG108. Data represent mean ± SD. (C) Percentages of ALB and AAT positive cells was performed by FACS analysis in hESC-Hep treated with DMSO or SB. (TIF) [file pone.0132992.s008.tif]

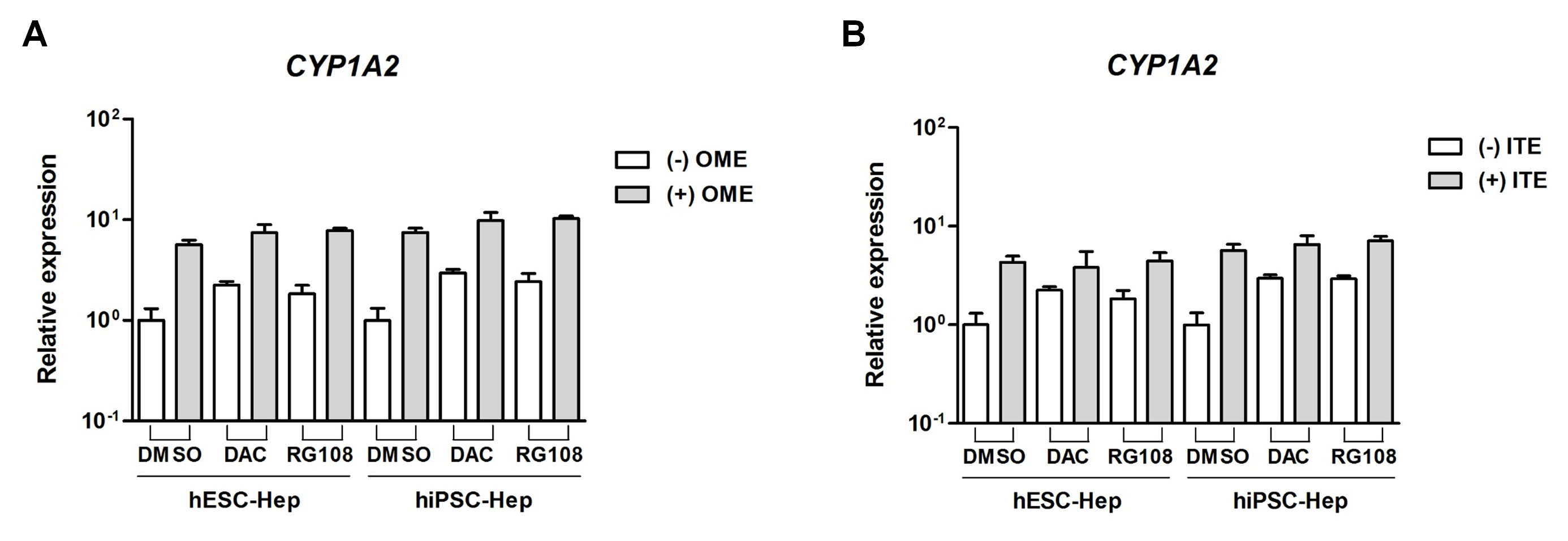

Supplement: S9 Fig — Expression level of CYP1A2 gene was examined by real-time RT-PCR in hESC-Hep (A) and hiPSC-Hep (B) treated with DMSO, DAC, or RG108 at day 15 of differentiation for 5 days and then further treated with 100 μM OME (omeprazole) or 0.5 μM ITE at day 19 of differentiation for 24 hr. Data represent mean ± SD. (TIF) [file pone.0132992.s009.tif]
