# Supplementary material for: Differences in the Epigenetic Regulation of Cytochrome P450 Genes between Human Embryonic Stem Cell-Derived Hepatocytes and Primary Hepatocytes
Source: PLoS One. 2015 Jul 15;10(7):e0132992. doi: 10.1371/journal.pone.0132992 (PMC4503736; doi:10.1371/journal.pone.0132992)
Supplement: S1 Table — (DOCX) [file pone.0132992.s010.docx]

**S1 Table. Primers used for real-time RT-PCR and RT-PCR analysis**

| **Gene** | **NCBI No.** | **Forward primer** | **Reverse primer** | **Product size (bp)** |
| --- | --- | --- | --- | --- |
| *AHR* | NM_001621.4 | caaatccttccaagcggcata | cgctgagcctaagaactgaaag | 123 |
| *NR1I3* | NM_001077470.1 | ctgtcggcagaagccctggc | gcccccaggagtgtccggat | 116 |
| *NR1I2* | NM_003889.3 | tgcgagatcacccggaagac | atgggagaaggtagtgtcaaagg | 255 |
| *NFE2L2* | NM_001145413.2 | tcagcgacggaaagagtatga | ccactggtttctgactggatgt | 174 |
| *CYP1A1* | NM_000499.3 | aggcttttacatccccaagg | ttgtcgatagcaccatcagg | 132 |
| *CYP1B1* | NM_000104.3 | acgtaccggccactatcact | ctccccacgacctgatcca | 142 |
| *CYP1A2* | NM_000761.3 | atgggcaagcgccggtgtat | cagttgatggagaagcgcagcc | 194 |
| *CYP2D6* | NM_000106.5 | ggtgtgacccatatgacatcc | ttggtgatgagtgtcgttcc | 77 |
| *CYP2E1* | NM_000773.3 | aggcaggtgcacagcagctg | tcaccaccatgcgctgcgag | 169 |
| *CYP2B6* | NM_000767.4 | ccggggatatggtgtgatctt | ccgaagtccctcatagtggtc | 84 |
| *CYP2C9* | NM_000771.3 | tgaagaagagcagatggcctg | agatgacaggtgagaaaaggca | 110 |
| *CYP2C19* | NM_000769.1 | aaaaccaaggcttcaccctgt | cccgggaaataatcaatgatagtgg | 212 |
| *CYP3A4* | NM_017460.5 | gtggggcttttatgatggtca | gcctcagatttctcaccaacaca | 272 |
| *CYP7A1* | NM_000780.3 | gagaaggcaaacgggtgaac | ggattggcaccaaattgcaga | 81 |
| *UGT1A1* | NM_000463.2 | ggaatcaactgccttcaccaa | acaggactgtctgagggattttg | 175 |
| *UGT1A6* | NM_001072.3 | gcttgaatatcctaggccggt | ggcttcaaattcctgagacaag | 94 |
| *UGT2B7* | NM_001074.2 | caagcattgcattgcaccagg | caatggctgtattctgctgcc | 128 |
| *SULT1A1* | NM_001055.3 | agaacaaccctgcattcccc | caggtagtgccggacttgg | 247 |
| *GSTA1* | NM_145740.3 | tccttctgcccgtatgtccacct | actggagtcaagctcctcgacgt | 200 |
| *GSTM1* | NM_000561.3 | atatgcagctgggcatgatct | gctcaaatatacggtggaggt | 192 |
| *GSTP1* | NM_000852.3 | aagccttttgagaccctgct | ggttcacgtactcaggggag | 223 |
| *ABCB1* | NM_000927.4 | gggagcttaacacccgactta | gccaaaatcacaagggttagctt | 154 |
| *ABCC2* | NM_000392 | tgagcaagtttgaaacgcacat | agctcttctcctgccgtctct | 78 |
| *ABCC3* | NM_001144070.1 | caccaactcagtcaaacgtgc | gcaagaccatgaaagcgactc | 197 |
| *SLC22A1* | NM_153187.1 | atcctgcacatgggcgccac | gcccacgcggtcaatggtga | 111 |
| *ABCB11* | NM_003742.2 | acgttgtgggttgctgaaca | ctgcggcaatgacccaaaag | 122 |
| *ALB* | NM_000477 | gagaccagaggttgatgtgatg | agttccggggcataaaagtaag | 114 |
| *AAT* | NM_000295 | gaagtcaaggacaccgagga | gctggcagaccttctgtctt | 261 |
| *AFP* | NM_001134 | agcttggtggtggatgaa | tctgcaatgacagcctcaag | 182 |
| *HNF4A* | NM_000457 | cgagcagatccagttcatca | tcacacatctgtccgttgct | 201 |
| *DNMT1* | NM_001130823 | agtccgatggagaggctaag | agcaactcgttctctggatgt | 266 |
| *DNMT3A* | NM_022552 | atggcaggatagccaagttc | ctttgccctgctttatggag | 72 |
| *DNMT3B* | NM_001207055 | ccgagaacaaatggcttcag | tcctgccacaagacaaacag | 74 |
| *HDAC1* | NM_004964 | aagccaatgctgaggagatg | aacaggccatcgaatactgg | 148 |
| *HDAC2* | NM_001527 | aggaggtcgaagaaatgtgg | ttcaccactgttgtccttgg | 133 |
| *HDAC3* | NM_003883 | accttttccagccggttatc | tcggatgctgaggttaaagc | 122 |
| *SIRT1* | NM_001142498 | cagtgtcatggttcctttgc | atagcaagcggttcatcagc | 137 |
| *SIRT2* | NM_001193286 | aggccatctttgagatcagc | agatggttggcttgaactgc | 96 |
| *SIRT3* | NM_001017524 | gaagctcatggaacctttgc | agaacacaatgtcgggcttc | 145 |
| *OCT4* | NM_002701 | gtactcctcggtccctttcc | caaaaaccctggcacaaact | 168 |
| *SOX2* | NM_003106 | cccagcagacttcacatgt | cctcccatttccctcgtttt | 151 |
| *CMYC* | NM_002467 | aagaggacttgttgcggaaa | ctcagccaaggttgtgaggt | 179 |
| *KLF4* | NM_004235 | gaactgaccaggcactaccg | ttctggcagtgtgggtcata | 152 |
| *REX1* | NM_174900 | cagatcctaaacagctcgcagaat | gcgtacgcaaattaaagtccaga | 306 |
| *TERT* | NM_198253 | tgtgcaccaacatctacaag | gcgttcttggctttcaggat | 166 |
| *ECAT15* | NM_018189 | ggagccgcctgccctggaaaattc | tttttcctgatattctattcccat | 408 |
| *GAPDH* | NM_002046 | catgagaagtatgacaacagcct | agtccttccacgataccaaagt | 113 |
