# Supplementary material for: Differences in the Epigenetic Regulation of Cytochrome P450 Genes between Human Embryonic Stem Cell-Derived Hepatocytes and Primary Hepatocytes
Source: PLoS One. 2015 Jul 15;10(7):e0132992. doi: 10.1371/journal.pone.0132992 (PMC4503736; doi:10.1371/journal.pone.0132992)
Supplement: S2 Table — (DOCX) [file pone.0132992.s011.docx]

**S2 Table. Primers used for bisulfite sequencing**

| **Gene** | **Primer sequence** | **Genomic region**  **(TSS = +1)** | **No. of CpGs** | **Product size (bp)** |
| --- | --- | --- | --- | --- |
| *CYP1A1* | F: tttggaaattttgtaataggaaggtt  R: acatccctctaaaaaacaaaaatca | −1353 to −898 | 42 | 458 |
| *CYP1B1* | F: ttgggattataggtgtgtattatta  R: accaaaaacaaccctacactttaaa | −1791 to −1362 | 17 | 430 |
|  | F: ttttattgaggtggtaatttgtttg  R: ctctaccaacaaactttcataaaaac | −435 to −123 | 26 | 313 |
| *CYP1A2* | F: tgttattttttgtttggtattttgg  R: tcaaaaccttaacctccttactcac | +950 to +1413 | 20 | 464 |
|  | F: tttttttaagtaaggataaatttttgagtt  R: aaaatatcataatccccacaacct | −1215 to −794 | 10 | 422 |
| *CYP2D6* | F: ttgtaggttttaggagtttggagtg  R: aaaatccacatacaacaaattaccc | −240 to +243 | 12 | 484 |
|  | F: tttttttagtgtaggtggttttttg  R: cctaaccctccctctacaattac | +954 to +1296 | 32 | 343 |
| *CYP2E1* | F: ttagggagaataatttagtaattggattt  R: taacccattcaatattcacaacaat | +232 to +629 | 23 | 398 |
| *OCT4* | F1 : ataaagtgagattttgttttaaaaa  R1 : aacataaaaaaatcccccacac  F2 : gggatttgtattgaggttttgg  R2 : cccacacctcaaaacctaac | -201 ~ +231 | 16 | 433 |
| *REX1* | F: ggtttaaaagggtaaatgtgattatattta  R: caaactacaaccacccatcaac | -428 ~ -68 | 35 | 361 |
| *NANOG* | F: tggttaggttggttttaaatttttg  R: aacccacccttataaattctcaatta | -387 ~ -52 | 8 | 336 |

F, forward; R, reverse; TSS, transcription start site.
