# Supplementary material for: Differences in the Epigenetic Regulation of Cytochrome P450 Genes between Human Embryonic Stem Cell-Derived Hepatocytes and Primary Hepatocytes
Source: PLoS One. 2015 Jul 15;10(7):e0132992. doi: 10.1371/journal.pone.0132992 (PMC4503736; doi:10.1371/journal.pone.0132992)
Supplement: S3 Table — (DOCX) [file pone.0132992.s012.docx]

**S3 Table. Primers used for chromatin immunoprecipitation**

| **Gene** | **Primer sequence** | **Genomic region**  **(TSS = +1)** | **Product size (bp)** |
| --- | --- | --- | --- |
| *CYP1A1* | F: agctaggccatgccaaatg  R: gcgattgaataaggggatgc | −1296 to −1150 | 147 |
|  | F: cggtccttctcacgcaac  R: atccctctagggggcagag | -914 to -827 | 87 |
| *CYP1B1* | F: catttgggcctcttatctgc  R: gcagccatcaagaagtggag | −1583 to −1488 | 104 |
|  | F: ttgtaccgagcgtggttct  R: acgtttccattgtgcggta | -243 to -157 | 86 |
| *CYP1A2* | F: catcttctgcctggtattctgg  R: gtggacttttcaggcctttg | +954 to +1024 | 71 |
| *CYP2D6* | F: cagaggagcccatttggtag  R: gcatcaggtccaccaggag | +62 to +161 | 100 |
| *CYP2E1* | F: gggtccagaaccttgattcc  R: tcacgtcccttccctcag | +776 to +846 | 71 |

F, forward; R, reverse; TSS, transcription start site.
