# Supplementary material for: Differences in the Epigenetic Regulation of Cytochrome P450 Genes between Human Embryonic Stem Cell-Derived Hepatocytes and Primary Hepatocytes
Source: PLoS One. 2015 Jul 15;10(7):e0132992. doi: 10.1371/journal.pone.0132992 (PMC4503736; doi:10.1371/journal.pone.0132992)
Supplement: S1 Materials and Methods — (DOCX) [file pone.0132992.s013.docx]

**Supporting Materials and Methods**

**CXCR4 FACS**

Cells were incubated with Accutase (Innovative Cell Technologies, San Diego, CA, USA) at 37°C for 10 minutes. Dissociated cells were washed with PBS containing 1% FBS and incubated for 1 hour with CXCR4 antibody. Cells were also labeled with the isotype control as a negative control. Primary antibody used for FACS is phycoerythrin (PE) mouse anti-human CD 184 (CXCR4) and isotype control is PE mouse IgG2a κ Isotype (BD Biosciences, San Jose, CA, USA). Flow cytometry was performed using BD FACS Calibur (BD Biosciences).

**Culture of hiPSCs**

Human iPSCs (provided by PhD. Yong-Mahn Han, KAIST) were derived from human dermal fibroblasts via the ectopic expression of OCT4, SOX2, KLF4, and c-MYC as previously described [[1](#_ENREF_1)]. hiPSCs were maintained on mitomycin C (MMC)-treated mouse embryonic fibroblasts in Dulbecco's modified Eagle medium (DMEM)/F-12 (Invitrogen, Carlsbad, CA) supplemented with 20% Knockout Serum Replacement (Invitrogen), 0.1 mM β-mercaptoethanol (Sigma-Aldrich, St. Louis, MO), 1% non-essential amino acids (Invitrogen), 1% penicillin-streptomycin (Invitrogen), and 10 ng/ml basic fibroblast growth factor (FGF; R&D Systems, Minneapolis, MN) at 37°C, 5% CO_2_ in air. The medium was changed daily.

**Characterization of hiPSCs**

**Immunofluorescence**

Cells were fixed in 4% formaldehyde (Sigma-Aldrich) for 30 minutes at RT, rinsed three times in PBS containing 0.1% Tween 20 (PBST) for 10 minutes, permeabilized in PBS containing 0.1% Triton X-100 (Sigma-Aldrich) for 15 minutes, and blocked for 1 hour in PBS containing 5% normal serum (Jackson ImmunoResearch, West Grove, PA, USA). Cells were incubated overnight at 4°C with the following primary antibodies diluted in PBS containing 5% normal serum: goat anti-OCT4 (1:100; Santacruz), rabbit anti-SOX2 (1:200; Cell signaling); mouse anti-SSEA4 (1:200; Abcam), mouse anti-TRA-1-60 (1:200; Millipore). Cells were rinsed six times in PBST for 10 minutes each. Thereafter, cells were incubated for 1 hour at RT with appropriate secondary antibodies (Alexa Fluor 488 or 594; Invitrogen) diluted in PBST (1:200). Cells were washed six times in PBST, and mounted in 4'-6-diamidino-2-phenylindole (DAPI, Sigma-Aldrich).

**RT-PCR**

Total RNA was isolated from cells using TRIzol Reagent (Invitrogen) and reverse-transcribed using SuperScript II Reverse Transcriptase (Invitrogen) according to the manufacturer’s protocol. The RT-PCR reaction was performed using following cycle condition: 95˚C denaturation for 30 sec, 60˚C annealing for 30 sec, 72˚C elongation for 30 sec and cycle numbers for each reaction varied between 30~35. GelRed (Biotium) was used for visualization of PCR product in gel electrophoresis. Primers used in this experiment are listed in S1 Table.

**Teratoma formation**

hiPSCs (1 X 10^7^ cells) were collected by scraping, mixed with matrigel, and subcutaneously injected into dorso-lateral area of CAnN.Cg-Foxn1 nu/Crljori mice (Orient, Seongnam, Korea). Around two months after injection, tumor tissues were dissected and embedded in paraffin wax. Tissue sections were placed on slide glasses. Hematoxylin and eosin (H&E, Sigma) staining was performed to observe various cell types and tissues.

**Reference**

1. Takahashi K, Tanabe K, Ohnuki M, Narita M, Ichisaka T, Tomoda K, et al. (2007) Induction of pluripotent stem cells from adult human fibroblasts by defined factors. Cell 131: 861-872.
